# Immunobiotic *Lactobacillus jensenii* TL2937 Alleviates Dextran Sodium Sulfate-Induced Colitis by Differentially Modulating the Transcriptomic Response of Intestinal Epithelial Cells

**DOI:** 10.3389/fimmu.2020.02174

**Published:** 2020-09-17

**Authors:** Nana Sato, Valeria Garcia-Castillo, Mao Yuzawa, Md. Aminul Islam, Leonardo Albarracin, Mikado Tomokiyo, Wakako Ikeda-Ohtsubo, Apolinaria Garcia-Cancino, Hideki Takahashi, Julio Villena, Haruki Kitazawa

**Affiliations:** ^1^Food and Feed Immunology Group, Graduate School of Agricultural Science, Tohoku University, Sendai, Japan; ^2^Livestock Immunology Unit, International Education and Research Center for Food Agricultural Immunology (CFAI), Graduate School of Agricultural Science, Tohoku University, Sendai, Japan; ^3^Laboratory of Bacterial Pathogenicity, Faculty of Biological Sciences, University of Concepcion, Concepción, Chile; ^4^Department of Medicine, Faculty of Veterinary Science, Bangladesh Agricultural University, Mymensingh, Bangladesh; ^5^Laboratory of Immunobiotechnology, Reference Center for Lactobacilli (CERELA-National Council for Scientific and Technological Research), San Miguel de Tucumán, Argentina; ^6^Laboratory of Computing Science, Faculty of Exact Sciences and Technology, Tucuman University, San Miguel de Tucumán, Argentina; ^7^Laboratory of Plant Pathology, Graduate School of Agricultural Science, Tohoku University, Sendai, Japan; ^8^Plant Immunology Unit, International Education and Research Center for Food Agricultural Immunology, Graduate School of Agricultural Science, Tohoku University, Sendai, Japan

**Keywords:** immunobiotics, intestinal inflammation, *Lactobacillus jensenii* TL2937, PIE cells, immunotranscriptomic response

## Abstract

Immunobiotics have emerged as a promising intervention to alleviate intestinal damage in inflammatory bowel disease (IBD). However, the beneficial properties of immunobiotics are strain dependent and, therefore, each strain has to be evaluated in order to demonstrate its potential application in IBD. Our previous *in vitro* and *in vivo* studies demonstrated that *Lactobacillus jensenii* TL2937 attenuates gut acute inflammatory response triggered by Toll-like receptor 4 activation. However, its effect on colitis has not been evaluated before. In this work, we studied whether the TL2937 strain was able to protect against the development of colitis in a dextran sodium sulfate (DSS)-induced mouse model and we delved into the mechanisms of action by evaluating the effect of the immunobiotic bacteria on the transcriptomic response of DSS-challenged intestinal epithelial cells. *L. jensenii* TL2937 was administered to adult BALB/c mice before the induction of colitis by the administration of DSS. Colitis and the associated inflammatory response were evaluated for 14 days. Mice fed with *L. jensenii* TL2937 had lower disease activity index and alterations of colon length when compared to control mice. Reduced myeloperoxidase activity, lower production of pro-inflammatory (TNF-α, IL-1, CXCL1, MCP-1, IL-15, and IL-17), and higher levels of immunoregulatory (IL-10 and IL-27) cytokines were found in the colon of TL2937-treated mice. In addition, the treatment of porcine intestinal epithelial (PIE) cells with *L. jensenii* TL2937 before the challenge with DSS differentially regulated the activation of the JNK pathway, leading to an increase in epithelial cell integrity and to a differential immunotranscriptomic response. TL2937-treated PIE cells had a significant reduction in the expression of inflammatory cytokines (*TNF*-α, *IL-1*α, *IL-1*β, *IL-6, IL-15*), chemokines (*CCL2, CCL4, CCL8, CXCL4, CXCL5, CXCL9, CXCL10*), adhesion molecules (*SELE, SELL, EPCAM*), and other immune factors (*NCF1, NCF2, NOS2, SAA2*) when compared to control cells after the challenge with DSS. The findings of this work indicate that (a) *L. jensenii* TL2937 is able to alleviate DSS-induced colitis suggesting a potential novel application for this immunobiotic strain, (b) the modulation of the transcriptomic response of intestinal epithelial cells would play a key role in the beneficial effects of the TL2937 strain on colitis, and (c) the *in vitro* PIE cell immunoassay system could be of value for the screening and selection of new immunobiotic strains for their application in IBD.

## Introduction

Inflammatory bowel disease (IBD), which includes Crohn's disease (CD) and ulcerative colitis (UC), is characterized by a chronic inflammation of the gastrointestinal tract (1, 2). Complex interactions between the environment, the gut microbiota, and the mucosal immune system in genetic-susceptible hosts have been implicated in the development and progress of IBD (3). Human clinical trials and IBD animal models have established that the breakdown of the cytokine networks that regulate the epithelial barrier function, the interaction of microbes with the immune system, and tissue repair are key players in the development of IBD (4). The great progress in the understanding of the intestinal cytokine networks that either suppress or promote gut inflammation has allowed the development of several preventive or therapeutic strategies based on cytokine regulation that are effectively used or are being evaluated in the clinic [reviewed in (2)]. Both the enhancement of immunoregulatory cytokines to prevent inflammation and the blockade of pro-inflammatory cytokine pathways have been shown to be effective in the treatment of IBD (1, 2).

Molecular and genomic studies in mouse models and in large IBD patient cohorts are revealing the role of several cytokine and their cellular pathways in the intestinal inflammatory damage, and therefore, many potential alternative cytokine-based therapies are being explored. Of interest, most of these cytokine-based therapies have focused their attention in the modulation of one single factor (1). The therapies based on the blockade of tumor necrosis factor (TNF)-α, which is effective in the majority of the IBD patients, is currently used as a standard therapy in the clinic (2). In patients with no response to TNF-α treatment, the blockade of other pro-inflammatory cytokines was effective in controlling inflammation. In this regard, the blockade of interleukin (IL)-6 (5), IL-1, or IL-18 (6, 7) is able to ameliorate colitis in IBD patients. Of note, despite the extensive research demonstrating the role of IL-17 and interferon (IFN)-γ in promoting inflammation in the gut of IBD patients or the protective role of IL-10, the blockade of IL-17A (8) or IFN-γ (9) as well as the systemic administration of IL-10 (10, 11) has not been beneficial in IBD. In addition, considering that most cytokines have a plethora of different roles and that the mentioned treatments are administered systemically, several non-desired side effects have been described for single cytokine-based therapies (1, 2). Perhaps the best example is the blockade of IL-6 that was described to induce gastrointestinal abscesses and perforation because of the key role of this cytokine in intestinal epithelial cells (IECs) repair (5).

The delicate balance between the beneficial and detrimental effects of cytokines as well as the complex interactions that are established between them in the context of IBD makes it necessary that the cytokine-based therapies are tightly controlled in a spatial, temporal, and quantitative manner. Then, therapies that can modulate the profile of several cytokines simultaneously, and that impact locally in the intestinal mucosa, would be potentially more effective and present fewer adverse effects. In this regard, extensive research has demonstrated that beneficial microbes with immunomodulatory capacities (immunobiotics) are able to differentially regulate intestinal cytokine profiles and, therefore, to protect against the inflammatory damage in both animal models and IBD patients (12, 13). Some of the most widely studied immunobiotic strains such as *Lactobacillus casei* Shirota, *L. casei* BL23, and *Escherichia coli* Nissle 1917 have been shown to exert beneficial effects in animal models of IBD by regulating cytokine profiles (12). On the other hand, other well-characterized immunobiotic strains including *L. acidophilus* NCFM (14) or *L. rhamnosus* GG (15) did not attenuate symptoms. Furthermore, some probiotics such as *L. crispatus* M206119 have been found to aggravate dextran sulfate sodium (DSS)-induced colitis in mice (16). These findings highlight the importance of accurately evaluating each immunobiotic candidate to be used in IBD.

*Lactobacillus jensenii* TL2937 is an immunobiotic strain that has been widely characterized by our group. Studies in porcine intestinal epithelial (PIE) cells demonstrated the ability of the TL2937 strain to inhibit nuclear factor κB (NF-κB) and mitogen-activated protein kinase (MAPK) signaling pathways (17), and to differentially regulate the expression levels of inflammatory cytokines and chemokines (18) in the context of Toll-like receptor (TLR)-4 activation. In addition, the TL2937 strain is capable of regulating the expression of activation markers and cytokine production in antigen-presenting cells from porcine Peyer's patches after the activation of TLR4 (19, 20). These *in vitro* findings prompted us to evaluate the immunoregulatory effects of *L. jensenii* TL2937 *in vivo*. Therefore, by using a porcine model we showed that the TL2937 strain improved the growing performance and the productivity of piglets by reducing the intestinal inflammation associated with weaning (21). Considering the remarkable ability of *L. jensenii* TL2937 to reduce inflammatory cytokines and to increase IL-10 in our *in vitro* and *in vivo* models, we hypothesized that this strain could exert beneficial effects in IBD. In this work, we studied whether the TL2937 strain was able to protect against the development of colitis in a DSS-induced mouse model. In addition, we delved into the mechanisms of action by evaluating the effect of the immunobiotic bacteria on the transcriptomic response of DSS-challenged intestinal epithelial cells.

## Materials and Methods

### Animals and Ethical Statement

Five-week-old female Balb/c mice were obtained from the closed colony at CERELA (Tucumán, Argentina). Animals were housed in plastic cages and environmental conditions were kept constant, in agreement with the standards for animal housing. All efforts were made to minimize the number of animals and their suffering. Animals were euthanized immediately after the time point was reached. No deaths were observed before mice reached the endpoints.

This study was carried out in strict accordance with the recommendations in the Guide for the Care and Use of Laboratory Animals of the Guidelines for Animal Experimentation of CERELA. The CERELA Institutional Animal Care and Use Committee prospectively approved this research under the protocol BIOT-IBT4-18.

### Immunobiotic Administration and Induction of Colitis

The immunobiotic strain *L. jensenii* TL2937 was grown in MRS medium (Difco, Detroit, MI) for 16 h at 37°C (17, 19). Cultures were kept freeze-dried and then rehydrated using the following medium: tryptone, 10.0 g; meat extract, 5.0 g; peptone, 15.0 g; and distilled water, 1 L, pH 7. Bacteria were cultured for 12 h at 37°C (final log phase) in Man–Rogosa–Sharpe broth (MRS, Oxoid, Cambridge, UK). Lactobacilli were harvested through centrifugation at 3,000 × g for 10 min and washed 3 times with sterile 0.01 mol/L phosphate buffer saline (PBS), pH 7.2, and suspended in sterile 10% non-fat milk for administration to mice (22).

**Figure 1:**
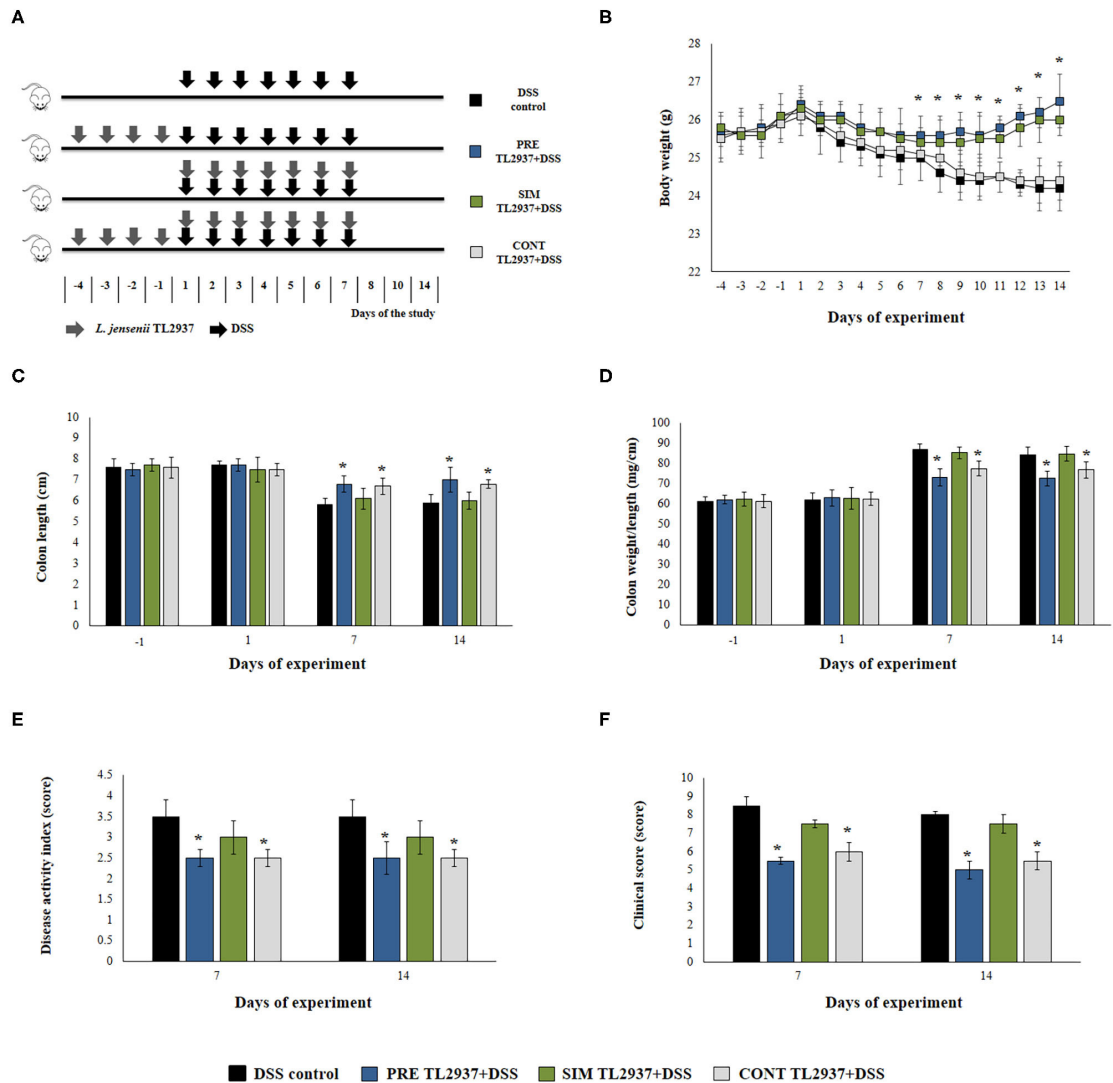
Effect of immunobiotic *Lactobacillus jensenii* TL2937 on the colitis induced in mice by dextran sodium sulfate (DSS) administration. **(A)** *L. jensenii* TL2937 was orally administered to different groups of mice (10^8^ cells/mouse/day) before (PRE DSS+TL2937 group), simultaneously (SIM DSS+TL2937 group), or continuously (CONT DSS+TL2937 group) with the administration of DSS for 7 days. Untreated mice challenged only with DSS were used as controls. The animals were sacrificed at different time points: the end of immunobiotic administration (day −1), the start of DSS administration (day 1), the end of DSS administration (day 7), and 7 days after the last DSS administration (day 14). **(B)** Body weight change was monitored daily during the study period. **(C)** Colon length, **(D)** colon length:weight ratio, **(E)** disease activity index, and **(F)** clinical scores were evaluated at the indicated days. The results represent three independent experiments. Significant differences when compared to the DSS control group: ^*^P < 0.05.

*L. jensenii* TL2937 was administered to different groups of mice before and/or during the induction of colitis as shown in Figure 1A. Mice were deprived of water for 4 h, and the immunobiotic strain was given at a dose of 10^8^ cells/mouse/day in a minimum volume of drinking water to animals in individual cages (22). Control mice were treated with water containing only non-fat milk. The lactobacilli-treated groups and the control mice were fed a conventional balanced diet *ad-libitum* during experiments.

Acute colitis was established using a previously established method (23). Briefly, mice were administered 2.5% (w/v) DSS 36–50 kDa (Thermo Fisher Scientific, USA) in drinking water *ad-libitum* for 7 days (Figure 1A). DSS was given to mice after 3 h of lactobacilli administration. Mice were examined daily for body weight, general appearance (piloerection and lethargy), stool consistency, and the presence of fecal blood. The animals were sacrificed at different time points (days −1, 1, 7, and 14, of Figure 1A) and fasted for 12 h prior to sacrifice.

### Disease Activity Index and Clinical Score

For the evaluation of the impact of *L. jensenii* TL2937 and DSS administration in the health of mice, two scores were calculated based in the observations performed by two different groups of qualified personnel: the disease activity index (DAI) (23, 24) and the clinical score (25). DAI was calculated considering the weight loss percentage, the presence of fecal blood, and stool consistency. DAI values were calculated as [(weight loss score) + (stool consistency) + (rectal bleeding score)]/4 and scored on a 0 ± 4 scale (Supplementary Table 1). The intensity of general appearance, weight loss, stool consistency, and rectal bleeding was graded to create a clinical score for the colitis ranging from 0 to 14 (Supplementary Table 1).

### Cytokine Concentrations

The determination of cytokine concentration in colon was performed following the method described by Rodrigues et al. (25). Tissue samples (100 mg) were collected from the distal portion of the colon and homogenized in 1 ml of PBS containing protease inhibitors (0.1 mM phenylmethylsulfonyl fluoride, 0.1 mM benzethonium chloride, 10 mM ethylenediaminetetraacetate, 20 KI aprotinin A, and 0.05% Tween 20) and centrifuged for 15 min at 3,000 × g. Blood samples were obtained by cardiac puncture under anesthesia as described previously (26). Intestinal fluid samples were obtained according to Albarracin et al. (27). Briefly, the small intestine was flushed with 5 ml of PBS and the fluid was centrifuged (10,000 g, 4°C for 10 min) to separate particulate material.

Tumor necrosis factor (TNF)-α, interleukin (IL)-1β, IL-6, IL-10, IL-15, IL-17, IL-27, transforming growth factor (TGF)-β, interferon (IFN)-γ, chemokine KC (or CXCL1), and monocyte chemoattractant protein-1 (MCP-1) concentrations in colon tissue were measured with commercially available mouse enzyme-linked immunosorbent assay (ELISA) technique kits following the manufacturer's recommendations (R&D Systems, MN, USA or Abcam, San Francisco, USA).

Caco-2 cell supernatants were collected after the treatments described below. TNF-α, IL-1β, IL-6, and IL-8 were measured with commercially available human ELISA technique kits following the manufacturer's recommendations (Abcam, San Francisco, USA).

### Myeloperoxidase (MPO) Assay in Colon Tissue

Neutrophil infiltration in the colon tissue was quantified indirectly by measurement of MPO. Colon sections were cleared of debris, washed in PBS, and weighed. Samples were homogenized in 50 mM acetate buffer, pH 5.4 (MPO-assay buffer). Homogenates were frozen at −70°C for 20 min, thawed, sonicated for 50 s, and centrifuged at 3,600 × g for 15 min at 4°C. MPO was evaluated by adding 200 ml of an appropriate dilution of the lysate to 20 mM 3,30,5,50-tetramethylbenzidine in dimethylformamide and 30 ml of 2.7 mM of hydrogen peroxide in MPO-assay buffer. The reaction mixture was incubated for 5 min at 37°C and stopped with ice-cold 200 mM sodium acetate buffer (pH 3) (26). Absorbance was read at 650 nm against a standard curve made with commercial MPO (Sigma). The results were expressed as specific activity of MPO (MPO units/g of colon homogenate).

### Human and Porcine Intestinal Epithelial Cells

The porcine intestinal epithelial (PIE) cell line was originally established by our group (28). PIE cells were isolated from intestinal epithelia derived from an unsuckled neonatal piglet. The PIE cells were maintained in Dulbecco's Modified Eagle's Medium (DMEM) enriched with 10% fetal calf serum (FBS), 100 U/ml penicillin, and 100 mg/ml streptomycin (Gibco, Thermo Fisher Scientific Co.) and plated into collagen (Type III)-coated 250 ml flask (Sumilon, Tokyo, Japan). After reaching confluence, the plate was washed twice with PBS and treated with the buffer containing 0.1 M Na_2_HPO_4_ 12 H_2_O, 0.45 M sucrose, 0.36% EDTA 4 Na, and BSA at 37°C for 5 min. Then, PIE cells were treated with a trypsin solution containing 0.25% trypsin and 0.02% EDTA in PBS at 37°C for 3 min, and the cells were collected by centrifugation (1,000 × g for 5 min at 4°C). After counting their numbers, PIE cells were seeded in a new flask, and after overnight culture, the supernatant was removed and fresh DMEM was added for a subsequent culturing. At least three consecutive passages were performed before the challenge experiments were conducted.

Human epithelial Caco-2 cells were grown in DMEM supplemented with 10% FBS, 50 U/ml penicillin, and 50 mg/ml streptomycin and maintained at 37°C in a humidified chamber of 5% CO_2_.

### Immunomodulatory Effect of *L. jensenii* TL2937 in PIE Cells

PIE cells were seeded at 3 × 10^4^ cells per well in 12-well type I collagen-coated plates (Sumitomo Bakelite Co., Tokyo, Japan) and cultured for 3 days as explained before. After changing the medium, *L. jensenii* TL2937 (5 × 10^7^ cells/ml) were added, and 48 h later, each well was washed vigorously with a medium at least three times to eliminate all stimulants. Then, cells were stimulated with DSS 5,000 Da (Wako Pure Chemical Industries Ltd., Osaka, Japan), dissolved in DMEM, and sterilized using DISMIC 25AS 0.45 μm (ADVANTEC, Tokyo, Japan). Different concentrations of DSS (0.1, 0.01, 0.001, and 0.0001%) were used for cytotoxicity assay (29). Viability was measured by a commercially available kit (Cell Titer 96™ AQueous, Promega, Madison, USA), which depends on the physiologic reduction of MTS to formazan. DSS 0.01% was used for the evaluation of transepithelial electrical resistance, western blot, qRT-PCR, and microarray studies. Caco-2 cells were seeded at 3 × 10^4^ cells per well in 12-well type I collagen-coated plates and treated with *L. jensenii* TL2937 as described for PIE cells. After 48 h, Caco-2 cells were stimulated with 1% of DSS (29) and inflammatory factors were evaluated by ELISA.

### Two-Step Real-Time Quantitative PCR (qRT-PCR)

Total RNA was isolated from each cell sample using TRIzol reagent (Invitrogen). Briefly, 100 μl of chloroform was added to samples and stirred. After 3 min standing, samples were centrifuged at 15,000 rpm, 15 min, 4°C. The upper layer (water layer) was collected and mixed with same amount of isopropanol. After standing for 10 min, samples were centrifuged at 15,000 rpm, 15 min, 20°C. The supernatants were eliminated, and the pellets were washed with 75% ethanol. After centrifugation (15,000 rpm, 15 min, 4°C), the supernatants were eliminated and pellets were dried at 50°C in a block incubator. Then, samples were diluted with DEPC water. Concentration and purity ware measured by a NanoDrop ND-1000 Spectrophotometer (NanoDrop Technologies, Inc., Wilmington, NC, USA).

DNAs were synthesized using a QuantiTect reverse transcription (RT) kit (Qiagen, Tokyo, Japan) according to the manufacturer's protocol. Real-time quantitative polymerase chain reaction (PCR) was performed with an Applied Biosystems Real-Time PCR System 7300 (Applied Biosystems, Warrington, United Kingdom) and the Platinum SYBR Green qPCR SuperMix-UDG (uracil-DNA glycosylase) with ROX (6-carboxyl-X-rhodamine) (Invitrogen). The primers for porcine immune factors, and β-actin is provided in Supplementary Table 2 or is described previously (18, 30). The PCR cycling conditions were 5 min at 50°C, followed by 5 min at 95°C, and then 40 cycles of 15 s at 95°C, 30 s at 60°C, and 30 s at 72°C. The reaction mixtures contained 2.5 μl of sample cDNA and 7.5 μl of master mix, which included the sense and antisense primers. According to the minimum information for publication of quantitative real-time PCR experiment guidelines, β-actin was used as a housekeeping gene because of its high stability across porcine various tissues (18, 30). Expression of porcine β-actin was used to normalize cDNA levels for differences in total cDNA levels in the samples.

The expressions of tight junctions and adherens junctions genes including occluding (*Ocln*), zonula occludens 1 (*ZO-1*), claudin 1 (*Cldn1*), and β-catenin (*Ctnn*) were evaluated by qRT-PCR in colon sections of mice. Tissue samples were cleared of debris, washed in sterile PBS, and weighed. RNA was extracted from the colon tissue samples using TRIzol reagent (Invitrogen, USA). The primers for murine tight junctions, adherens junctions, and β-actin are provided in Supplementary Table 2. The reaction mixtures contained 5 μl SYBR Premix Ex Taq™ II (5×), 0.2 μl Rox, 0.5 μl primer mix (10 μM), and 0.5 μl cDNA. The PCR cycling conditions were 30 s at 95°C, 40 cycles of 95°C for 5 s, and 60°C for 30 s. The expression of murine β-actin was used to normalize cDNA levels for differences in total cDNA levels in the samples.

### Assessment of Intestinal Epithelial Barrier Function

Transepithelial electrical resistance (TER) was used as a measure of barrier function in confluent PIE cell monolayers after the challenge with 5,000 Da DSS. For growth on porous filters, PIE cells were grown in the DMEM and plated at 1.0 × 10^6^ cells on a 0.4-μm PTFE membrane (Corning, NY, USA). Cellular TEERs were measured with an electrical resistance system, Millicell ERS-2 Voltohmmeter (Merck Millipore, Massachusetts, USA). Cells with stable TER readings >500 øcm were used (4–5 weeks post plating). To evaluate the stimulus effect on the epithelial barrier, TEER was measured at baseline and after 48 h of stimulation. The blank measurements (transwell without cell monolayer) were subtracted from TEER values of each experimental condition and were adjusted for the filter surface (0.3 cm^2^). The values were expressed as ohms per cm^2^. The results represent the percentage of final TER increases with respect to their basal TER value.

### Apoptosis Evaluation

The GFP-CERTIFIED Apoptosis/Necrosis detection kit (Enzo Life Sciences, Farmingdale, NY, USA) was used to evaluate apoptosis in DSS-challenged PIE cells. The cells (6.0 × 10^4^ cells/2 ml) were seeded into a Celtite C-1 Collagen-coated 6-well plate (SUMILON, Tokyo, Japan) and cultured at 37°C, 5% CO_2_. Apoptosis-positive controls were generated by the treatment of PIE cells with 1 mM of staurosporine for 4 h. After stimulations, 1 ml of epithelial buffer was added to each well and incubated for 3 min. Cells were detached by adding 500 μl of trypsin for 1 min and centrifugation at 800 rpm for 5 min. The cell pellets were washed with PBS, mixed with Dual Detection Regent, and kept at room temperature for 15 min in the dark. The measurement was performed with BD Accuri C6 Plus (BD, New Jersey, USA) and analyzed with FLOWJO (FLOWJO, Oregon, USA).

The caspase 3/7 was evaluated by using the CellEvent Caspase-3/7 Green Flow Cytometry Assay Kit (Thermo Fisher Scientific, Massachusetts, USA). PIE cells (6.0 × 10^4^ cells/2 ml) were treated with 1 μl of CellEvent Caspase-3/7 Green Detection Reagent and incubated at 37°C for 25 min in the dark. Then, 1 μl of SYTOX AADvanced reagent was added and incubated at 37°C for 5 min in the dark. The measurement was performed with BD Accuri C6 Plus (BD, New Jersey, USA) and analyzed with FLOWJO (FLOWJO, Oregon, USA).

### Western Blot Analysis

PIE cells were stimulated with *L. jensenii* TL2937 (5.1 × 10^8^ cells/well) for 48 h. Then, the cells were washed three times with DMEM medium to eliminate the bacteria and subsequently challenged with 0.1% of DSS for 0, 10, 20, 30, and 60 min. The PIE cells were washed and resuspended in 200 μl of CelLytic M Cell Lysis Reagent (Sigma-Aldrich, St. Louis, MO, USA), containing protease and inhibitors of phosphates (Complete mini, PhosSTOP, Roche, Mannheim, Germany). Cells were transferred to 1.5-ml Eppendorf tubes and kept at 95°C for 5 min in a water bath. The concentration of protein was estimated using BCA assay kit (Pierce, Rockford, IL). The lysed samples (8 μg/sample) were loaded on 10% SDS-polyacrylamide gels, and separated proteins were transferred electrophoretically to a nitrocellulose membrane. The cells were rinsed with PBS and then lysed in RIPA buffer (PBS, 1% Nonidet P-40, 0.5% sodium deoxycholate, and 0.1% SDS) with a protease inhibitor cocktail (Sigma Chemical Co.) at 4°C. Total cell lysates were separated with 8% SDS–polyacrylamide gel electrophoresis and transferred to PVDF membrane. Jun N-terminal protein kinase (JNK) was evaluated using anti-phosphated JNK antibodies (Santa Cruz Biotech). Blots were developed using ECL Western blotting detection reagent kit (Amersham Pharmacia Biotech, Inc., Piscataway, NJ). The optical protein bands were detected by ECF substrate (GE Healthcare Japan Co., Tokyo, Japan) and estimated from the peak area of densitogram by using ImageJ software (National Institutes of Health, Bethesda, MD, USA).

### Intracellular Ca^2+^ Flux in PIE Cells

Intracellular calcium mobilization was measured using the Fluo-4 Direct™ Calcium Assay Kits from Invitrogen according to the manufacturer's instructions (Dojindo, Kumamoto, Japan) as described previously (31). Briefly, PIE cells were plated in 96-well white-walled plates and grown to 90% confluence. Cells were serum-starved overnight and loaded with cell-permeant Fluo-4-AM diluted in calcium-free Hanks' balanced salt solution supplemented with 20 mm HEPES buffer provided by the manufacturer. Intracellular calcium flux was measured by fluorescence spectroscopy every 5 s for a total of 220 s (Victor2 Wallac, PerkinElmer Life Sciences). Background fluorescence was measured 30 s before addition of DSS and the average background subtracted from each value.

### Immunofluorescence

PIE cells (1.0 × 10^6^) were seeded on a Transwell 6.5-mm-inserted, collagen-coated 0.4-μm PTFE membrane (Corning, NY, USA) and cultured for 8 days. Then, PIE cells were stimulated with *L. jensenii* TL2937 (5 × 10^7^ cells/ml) for 48 h and challenged with 0.1% DSS for 6 h. The Transwell was washed with PBS and fixed with 4% paraformaldehyde phosphate buffer (room temperature, 20 min). Thereafter, the plate was washed with PBS and blocked with 2% goat serum (room temperature, 20 min). After washing with PBS-Tween, 50 μl of Can Get Signal Solution 1 was added to each well, and 1 μl of anti-ZO-1 or anti-β-catenin was added to make a 50-fold dilution. It was left overnight at 4°C, protected from light. After washing with PBS-Tween, 500 μl of Can Get Signal Solution 2 and 0.5 μl of Alexa Flour 488 were added (×1,000). The plate was allowed to stand for 2 h in the shade and washed with PBS-Tween. The membrane was excised with scissors for dissection, sealed with a mounting medium containing DAPI, dried overnight in a vat, and observed with OLYMPUS IX70 (OLYMPUS, Tokyo, Japan).

### RNA Isolation and Quality Control for Microarray Analysis

Total RNA was isolated from the ligand-treated and control PIE cells using PureLink RNA Mini Kit (Life Technology Inc., USA) along with on-column DNase treatment (18, 30). RNA integrity, quality, and quantity were evaluated with microcapillary electrophoresis (2100 Bioanalyzer, Agilent Technologies, Santa Clara, CA, USA) using the Agilent RNA 6000 Nano Kit (Agilent Technologies, Santa Clara, CA, USA). Only samples with an RNA integrity number (RIN) of >8 were used for this gene expression study.

### Microarray Hybridization

The microarray hybridization was performed with a Porcine Gene Expression Microarray 4 × 44K oligonucleotide slide (v2.0, Agilent Technologies, Santa Clara, CA, USA) containing 43,803 probes for the identification of known genes of the porcine transcriptome. The microarray experiment was conducted at Hokkaido System Science Co., according to the one-color Microarray-Based Gene Expression Analysis Protocol v6.7 (Agilent Technologies, Santa Clara, CA, USA). For each sample, 200 ng of total RNA was converted into cDNA by reverse transcription. The cDNA was subsequently transcribed into cRNA and labeled with cyanine 3 (Cy3). About 1.65 μg of labeled cRNA was mixed with hybridization buffer and hybridized on a microarray slide (4 samples in each slide) for 17 h at 65°C with constant rotation. After hybridization, microarrays were cleaned with Gene Expression Wash Buffer and scanned with High-Resolution Microarray Scanner (Agilent Technologies, Santa Clara, CA, USA). The Feature Extraction software (v10.7.3.1, Agilent Technologies, Santa Clara, CA, USA) was used for detailed analysis of scanned images including filtering the outlier spots, background subtraction from features, and dye normalization. The spot intensity data for individual sample were extracted for statistical analysis.

### Statistical Analysis of Microarray Data

The normalization and differential expression analysis of microarray data were performed with GeneSpring GX software (v13.1, Agilent Technologies, USA). The log_2_-transformed expression values of probes were normalized based on 75 percentile shifts. In order to determine the differential expression of genes, an unpaired *t*-test was performed between untreated control and stimulated samples. The pair-wise comparisons were performed between control and each of the stimulations to detect the differentially expressed genes. The Benjamini and Hochberg (B-H) adjustment method was applied for multiple-test correction. Significant differentially expressed genes were selected on the basis of two criteria: an adjusted *p*-value (FDR, false discover rate) of <0.05 and a cutoff in fold change of at least 1.5 (18, 30). The human ortholog gene symbol of DEGs was determined using the dbOrtho panel of the bioDBnet tool (32) which was used for downstream functional analysis.

### Network Enrichment Analyses

In order to visualize the *L. jensenii* TLR2937-mediated immunotranscriptional network in DSS-challenged PIE cells as well as to identify the regulatory genes, the subnetwork enrichment analysis was performed using the NetworkAnalyst online tool (33). This tool uses the InnateDB protein–protein interaction datasets composed of 14,755 proteins and 145,955 literature-curated interactions for humans (34). Human orthologous gene symbols of the common DEGs from all three stimulation were uploaded into the NetworkAnalyst to construct the interaction network based on the Walktrap algorithm taking only direct interaction of seed genes. The network was depicted as nodes (circles representing genes) connected by edges (lines representing direct molecular interactions). Two topological measures such as degree (number of connections to the other nodes) and betweenness (number of shortest paths going through the nodes) centrality were taken into account for detecting highly interconnected genes (Hubs) of the network. Nodes having a higher degree and betweenness were considered as potentially important Hubs in the cellular signal trafficking.

### Statistical Analysis

For *in vivo* experiments, each experimental group consisted of three mice per group at each time point and experiments were performed in triplicate (*n* = 9 for each parameter studied). Results were expressed as mean ± standard deviation (SD). The differences between groups were analyzed using the student *t*-test. Differences were considered significant at *P* < 0.05 and *P* < 0.01. ANOVA one-way was used for analysis of variance among multiple groups. For *in vitro* experiments, the statistical analysis was performed using GLM procedures of the SAS computer program. Mean values of relative mRNA expression were compared using the Bonferroni correction and multicomparison tests. Differences were considered significant at *P* < 0.05.

## Results

### *L. jensenii* TL2937 Alleviates DSS-Induced Colitis *in vivo*

We first aimed to evaluate whether the oral administration of the immunobiotic strain *L. jensenii* TL2937 was capable of avoiding or reducing the intestinal inflammatory damage induced by the DSS administration. For this purpose, three lactobacilli treatment schemes were assessed as shown in Figure 1A. The TL2937 strain was orally administered to mice before (PRE DSS+TL2937 group), simultaneously (SIM DSS+TL2937 group), or continuously (CONT DSS+TL2937 group) with the administration of DSS. The body weight change was used to evaluate the general health status of TL2937- and DSS-treated mice when compared with controls that received only DSS (Figure 1B). Similar to DSS control animals, mice in the SIM DSS+TL2937 group presented a gradual and continuous loss of body weight throughout the period studied. Mice in the PRE DSS+TL2937 and CONT DSS+TL2937 groups showed decreases in body weights that were similar to those observed in controls during the first 6 days after the administration of DSS. However, these groups of TL2937-treated mice started to recover body weights and showed values of this parameter that were significantly higher than controls from days 7 to 14 (Figure 1B). The colon length and the colon length:weight ratio were evaluated to determine the severity of colitis (Figures 1C,D). As expected, no significant differences were found in the values of these parameters when control mice and the TL2937-treated groups were compared before the administration of DSS (day −1). In addition, no differences between the experimental groups were detected on day 1 after DSS challenge. Similar to DSS control animals, mice in the SIM DSS+TL2937 group had reduced colon lengths and increased colon length:weight ratios on days 7 and 14 after DSS administration (Figures 1C,D). On the contrary, on days 7 and 14 the mice in the PRE DSS+TL2937 and CONT DSS+TL2937 groups showed significantly increased colon lengths and decreased colon length:weight ratios when compared to DSS controls (Figures 1C,D). To correlate the general health status with the colon damage, we further evaluated the disease activity index (DAI) and the clinical score on days 7 and 14 after DSS administration (Figures 1E,F). Both DAI and clinical score values were similar in the SIM DSS+TL2937 group and control DSS mice. However, the PRE DSS+TL2937 and CONT DSS+TL2937 groups had DAI and clinical score values that were significantly lower than the DSS control mice (Figures 1E,F). In particular, the absence of visible blood in rectum or on fur was evident in PRE DSS+TL2937 and CONT DSS+TL2937 mice.

### *L. jensenii* TL2937 Differentially Regulates the DSS-Triggered Inflammatory Response *in vivo*

**Figure 2:**
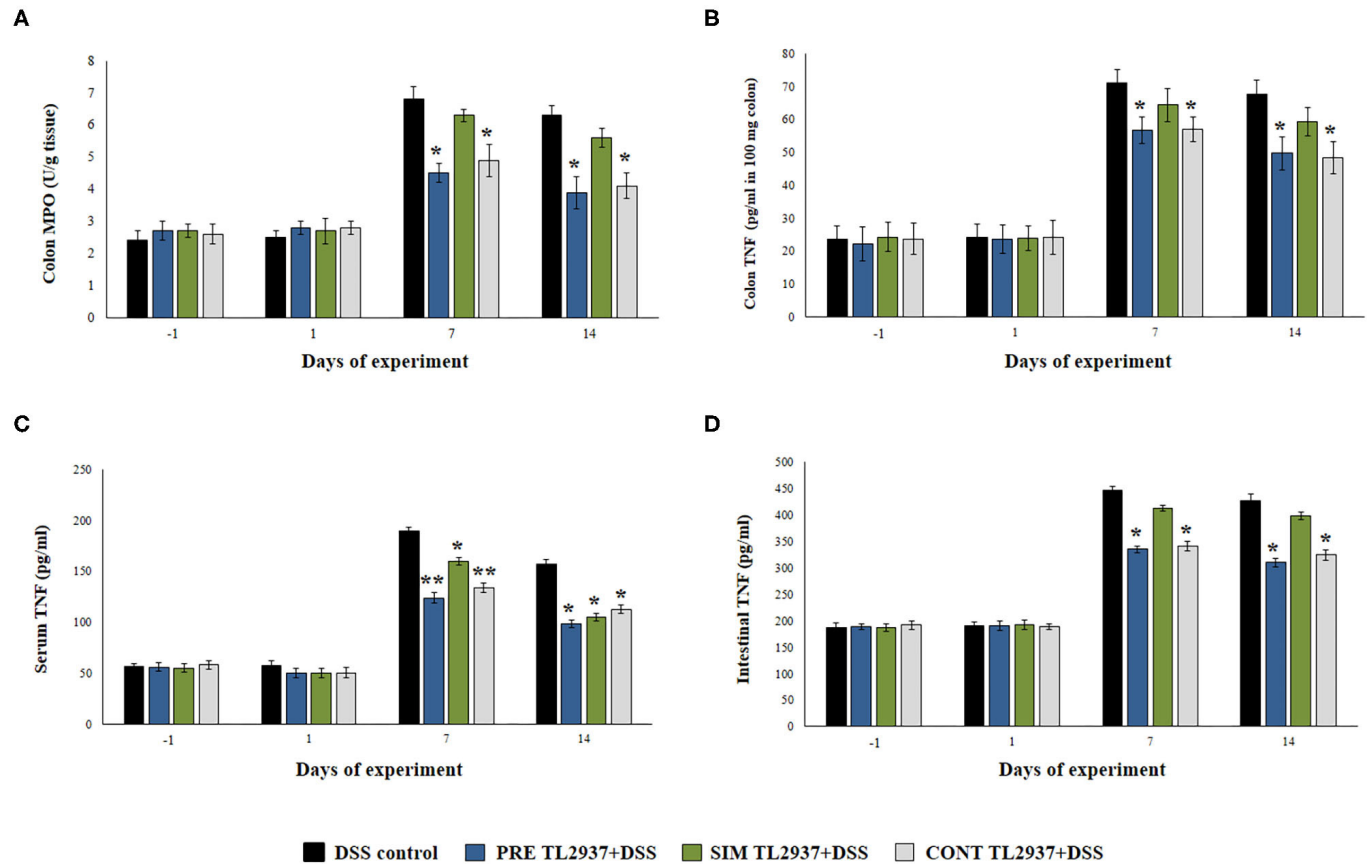
Effect of immunobiotic *Lactobacillus jensenii* TL2937 on the inflammatory response induced in mice by dextran sodium sulfate (DSS) administration. *L. jensenii* TL2937 was orally administered to different groups of mice (10^8^ cells/mouse/day) before (PRE DSS+TL2937 group), simultaneously (SIM DSS+TL2937 group), or continuously (CONT DSS+TL2937 group) with the administration of DSS for 7 days. Untreated mice challenged only with DSS were used as controls. The animals were sacrificed at different time points: the end of immunobiotic administration (day −1), the start of DSS administration (day 1), the end of DSS administration (day 7), and 7 days after the last DSS administration (day 14). **(A)** Colon myeloperoxidase (MPO) activity, **(B)** colon TNF-α, **(C)** serum TNF-α, and **(D)** small intestine TNF-α were evaluated at the indicated days. The results represent three independent experiments. Significant differences when compared to the DSS control group: ^*^P < 0.05, ^**^P < 0.01.

We next studied the effect of the immunobiotic treatments on the inflammatory response triggered by the administration of DSS. Since the increased production of TNF-α and the infiltration of neutrophils have been described as key factors in the inflammatory damage observed in DSS-induced colitis (1, 2), we evaluated the colon MPO activity as an indirect marker of neutrophil infiltration and the concentration of colon TNF-α (Figures 2A,B). In addition, the levels of serum (Figure 2C) and intestinal (Figure 2D) TNF-α were also determined. No significant differences were found in the values of these four parameters when control mice and the TL2937-treated groups were compared before DSS administration (day −1) or on day 1 after the DSS challenge. However, significantly higher levels of colon TNF-α and MPO activity as well as intestinal and serum TNF-α were found in all the experimental groups at days 7 and 14 after DSS administration when compared to day 1 (Figure 2). On days 7 and 14, no differences were observed between the SIM DSS+TL2937 group and control DSS mice. On the contrary, the PRE DSS+TL2937 and CONT DSS+TL2937 groups had significantly lower levels of colon MPO activity and TNF-α in colon, intestine, and serum than control DSS mice (Figure 2).

**Figure 3:**
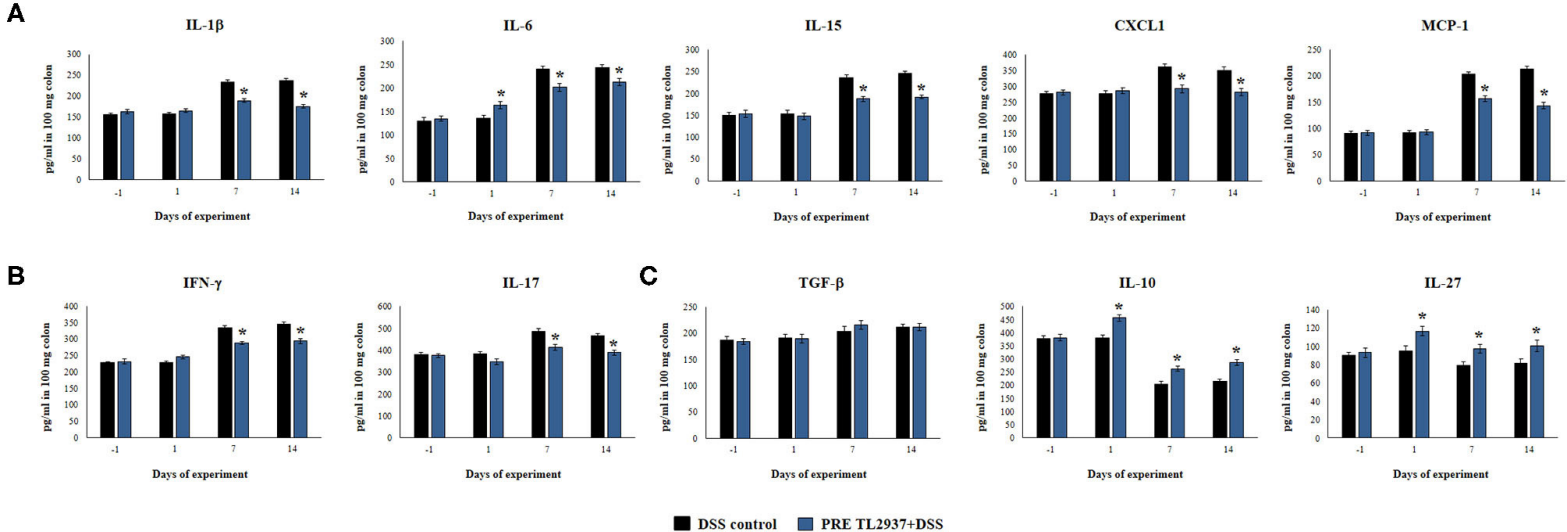
Effect of immunobiotic *Lactobacillus jensenii* TL2937 on the inflammatory response induced in mice by dextran sodium sulfate (DSS) administration. *L. jensenii* TL2937 was orally administered to different groups of mice (10^8^ cells/mouse/day) before (PRE DSS+TL2937 group) the administration of DSS for 7 days. Untreated mice challenged only with DSS were used as controls. The animals were sacrificed at different time points: the end of immunobiotic administration (day −1), the start of DSS administration (day 1), the end of DSS administration (day 7), and 7 days after the last DSS administration (day 14). **(A)** Colon inflammatory cytokines and chemokines (IL-1β, IL-6, CXCL1, IL-15, MCP-1), **(B)** colon IFN-γ and IL-17, and **(C)** colon regulatory cytokines (TGF-β, IL-10, IL-27) were evaluated at the indicated days. The results represent three independent experiments. Significant differences when compared to the DSS control group: ^*^P < 0.05.

Considering that *L. jensenii* TL2937 was able to differentially regulate the production of TNF-α in the colon and diminished the recruitment of neutrophils, we selected the PRE DSS+TL2937 treatment to further characterize the immunomodulatory activity of the TL2937 strain by assessing the levels of colon IL-1β, IL-6, IL-15, CXCL1, MCP-1, IFN-γ, IL-17, TGF-β, IL-10, and IL-27 (Figure 3). The oral administration of *L. jensenii* TL2937 to mice did not induce significant changes in the levels of the colon cytokines evaluated before DSS administration (day −1). In addition, no differences in the levels of colon pro-inflammatory cytokines were observed when the day −1 and the day 1 after DSS administration were compared, with the exception of IL-6 that were significantly higher in the PRE DSS+TL2937 group (Figures 3A,B). All the pro-inflammatory colon cytokines evaluated were increased on days 7 and 14 after DSS administration when compared to day 1 (Figures 3A,B). Interestingly, the concentrations of the cytokines IL-1β and IL-6 and the chemokines IL-15, CXCL1, and MCP-1 were significantly lower in the colon of PRE DSS+TL2937 mice than in the DSS control group (Figure 3A). The PRE DSS+TL2937 mice also had lower levels of colon IFN-γ and IL-17 than DSS controls (Figure 3B). When the immunoregulatory cytokines were analyzed, it was observed that there were no differences in the levels of colon TGF-β between days −1 and 1. On day 1, the levels of colon IL-10 and IL-27 were significantly higher in the PRE DSS+TL2937 group than in DSS controls (Figure 3C). A decrease in IL-10 and IL-27 was observed when days 7 and 14 were compared to day 1 in DSS control mice. In addition, it was found that IL-10 and IL-27 were significantly higher in the PRE DSS+TL2937 group than in the DSS control group on days 7 and 14 after the challenge with DSS while no differences between the groups were observed for colon TGF-β concentrations (Figure 3C).

**Figure 4:**
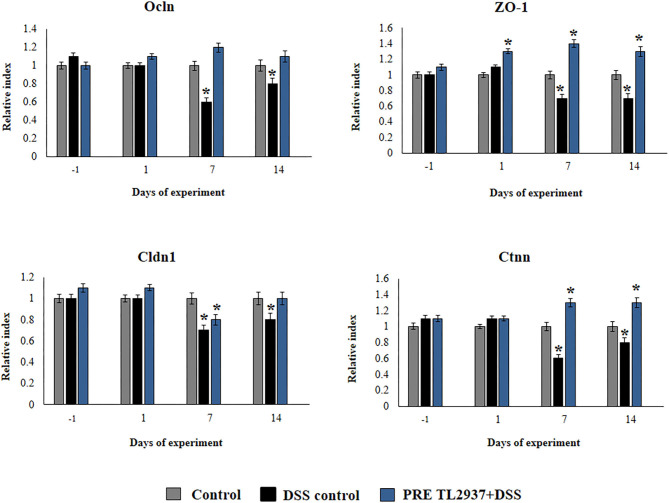
Effect of immunobiotic *Lactobacillus jensenii* TL2937 on the inflammatory response induced in mice by dextran sodium sulfate (DSS) administration. *L. jensenii* TL2937 was orally administered to different groups of mice (10^8^ cells/mouse/day) before (PRE DSS+TL2937 group) the administration of DSS for 7 days. Untreated mice challenged only with DSS were used as controls. The animals were sacrificed at different time points: the end of immunobiotic administration (day −1), the start of DSS administration (day 1), the end of DSS administration (day 7), and 7 days after the last DSS administration (day 14). The expressions of occluding (*Ocln*), zonula occludens 1 (*ZO-1*), claudin 1 (*Cldn1*), and β-catenin (*Ctnn*) were evaluated by qRT-PCR at the indicated days. The results represent three independent experiments. Significant differences when compared to the DSS control group: ^*^P < 0.05.

We also evaluated the effect of *L. jensenii* TL2937 in the expression of *Ocln, ZO-1, Cldn1*, and *Ctnn* in the colon of mice treated with DSS (Figure 4). No differences in the expression levels of *Ocln, ZO-1, Cldn1*, and *Ctnn* were observed when DSS controls and PRE DSS+TL2937 groups were compared to basal controls in days −1 and 1 after DSS administration, with the exception of *ZO-1* that was significantly enhanced in mice receiving the TL2937 strain. The DSS challenge reduced the expressions of *Ocln, ZO-1, Cldn1*, and *Ctnn* on days 7 and 14 after its administration. Of note, mice in the PRE DSS+TL2937 group had significantly higher levels of *Ocln, ZO-1*, and *Ctnn* on days 7 and 14 when compared to DSS controls. The only exception was *Cld1* that was not different when DSS controls and PRE DSS+TL2937 groups were compared on day 7 (Figure 4). While mice in the PRE DSS+TL2937 group had normal levels of *Ocln* and *Cldn1*, the levels of *ZO-1* and *Ctnn* were higher than basal controls in days 7 and 14 after DSS administration.

### *L. jensenii* TL2937 Diminishes DSS-Triggered Epithelial Barrier Alterations *in vitro*

**Figure 5:**
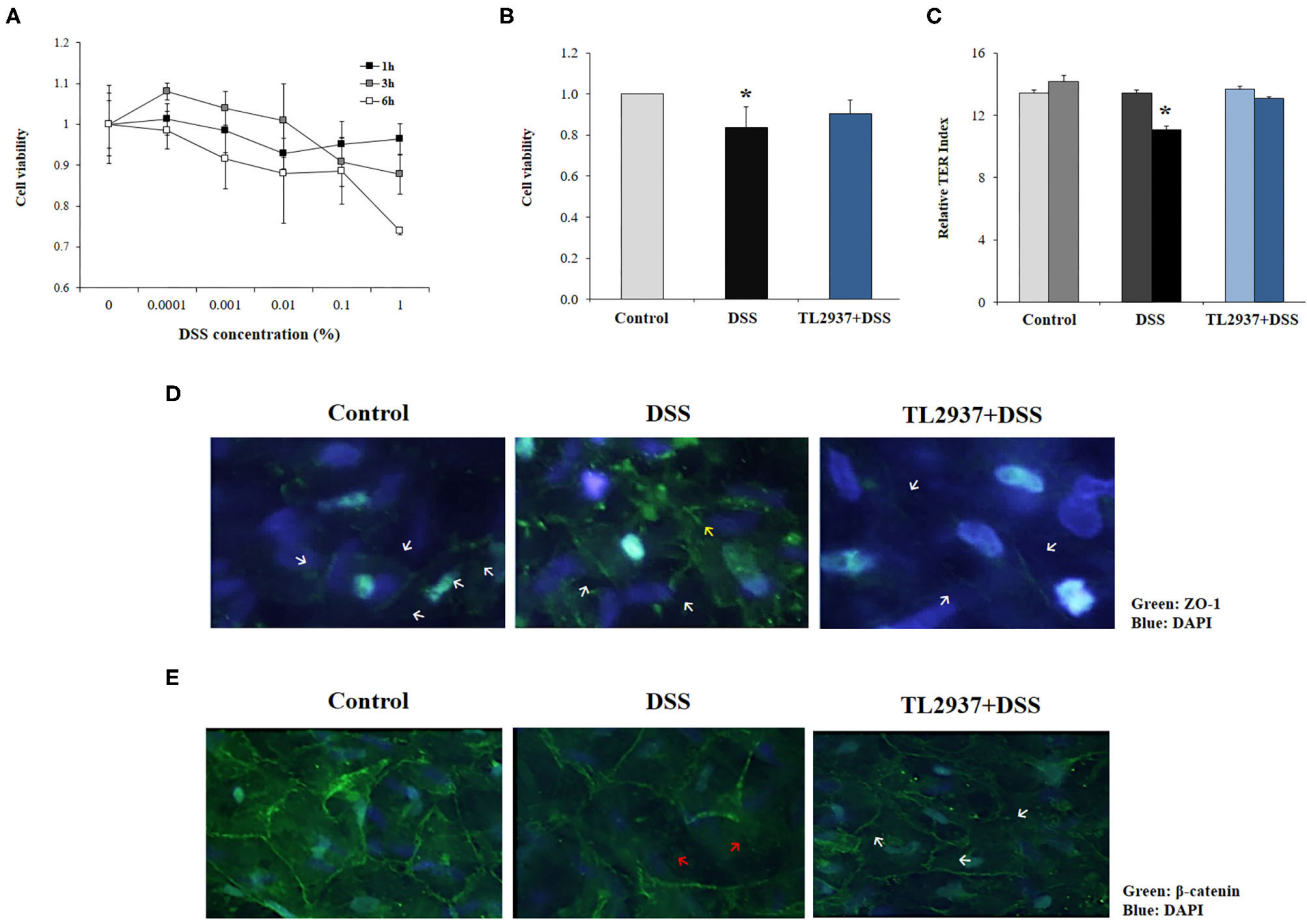
Effect of immunobiotic *Lactobacillus jensenii* TL2937 on the *in vitro* epithelial barrier alterations induced by dextran sodium sulfate (DSS) administration. Porcine intestinal epithelia (PIE) were challenged with different concentrations of DSS for 1, 3, or 6 h for evaluation of **(A)** cell viability. The dose of 0.01% DSS for 6 h was selected for further experiments. PIE cells were stimulated with *L. jensenii* TL2937 (5 × 10^7^ cells/ml) for 48 h and then challenged with DSS. PIE cells challenged only with DSS were used as controls. **(B)** Cell viability, **(C)** transepithelial electrical resistance (TER), **(D)** ZO-1 expression, and **(E)** β-catenin expression were determined. TER index values before and after DSS challenge are indicated with white and black bars, respectively. The results represent three independent experiments. Significant differences when compared to the unchallenged control group: ^*^P < 0.05.

Considering our previous studies demonstrating the ability of *L. jensenii* TL2937 to differentially modulate inflammatory responses in intestinal epithelial cells (17, 18), we hypothesized that its beneficial effect in DSS-colitis *in vivo* could be related to the modulation of the response of these cells to the challenge with DSS. Then, we next aimed to characterize the effect of *L. jensenii* TL2937 on intestinal epithelial cells in the context of DSS-induced inflammatory damage. For this purpose, we used as a model the PIE cell line developed by our group (28). We reported previously that PIE cells show epithelial-like morphology and create a monolayer attaching to neighboring cells (28, 35, 36). These characteristics of PIE cells give us the possibility of conducting *in vitro* studies to evaluate the epithelial barrier function. We first evaluated the response of PIE cell monolayers to the challenge with different concentrations of DSS during 1, 3, or 6 h in terms of their viability (Figure 5A). A dose-dependent effect was observed when the viability of PIE cells was assessed after the challenge with the different concentration of DSS. While no significant differences in cell viability were induced by 0.0001% of DSS, the higher concentration (1%) reduced PIE cell viability by 25%. No significant differences were found when the 1, 3, and 6 h of stimulation were compared within the same dose of DSS, with the exception of DSS 1% for 6 h that induced the highest cell mortality (Figure 5A). Considering those results, we selected 0.01% of DSS for 6 h for further studies.

To evaluate the effect of *L. jensenii* TL2937, PIE cells were treated with the bacteria, challenged with DSS and the viability (Figure 5B) and the TER (Figure 5C) were evaluated. TL2937-treated PIE cells had a significantly increased viability after the challenge with DSS when compared to controls (Figure 5B). The treatment of PIE cells with *L. jensenii* TL2937 did not induce modifications in the TER index (Figure 5C). On the contrary, DSS challenge diminished the TER index when compared to unchallenged cells. Interestingly, TL2937-treated PIE cells had normal TER index values after the stimulation with DSS (Figure 5C). The barrier function of mucosal epithelial cells is mediated mainly by the molecular structures that are established between neighboring cells such as tight junctions and adherens junctions. Then, we next evaluated the expression of ZO-1 (Figure 5D) and β-catenin (Figure 5E) in PIE cell monolayers to study tight junctions and adherens junctions, respectively. Immunofluorescence studies showed that both proteins are mainly expressed in the cell–cell contact areas. The treatment of PIE cells with DSS altered their expression of ZO-1 (Figure 5D) and β-catenin (Figure 5E). An increased cytoplasmic expression and an altered distribution on the cell surface of ZO-1 were observed in DSS control PIE cells while TL2937-treated cells showed a pattern of ZO-1 expression that was not different from unchallenged cells (Figure 5D). DSS stimulation reduced both cytoplasmic and cell surface expression of β-catenin. Although the same reduction in β-catenin expression was observed in TL2937-treated cells after DSS challenge, PIE cells in this group have some areas with accumulations of this protein both in the cytoplasm and on their surface (Figure 5E).

**Figure 6:**
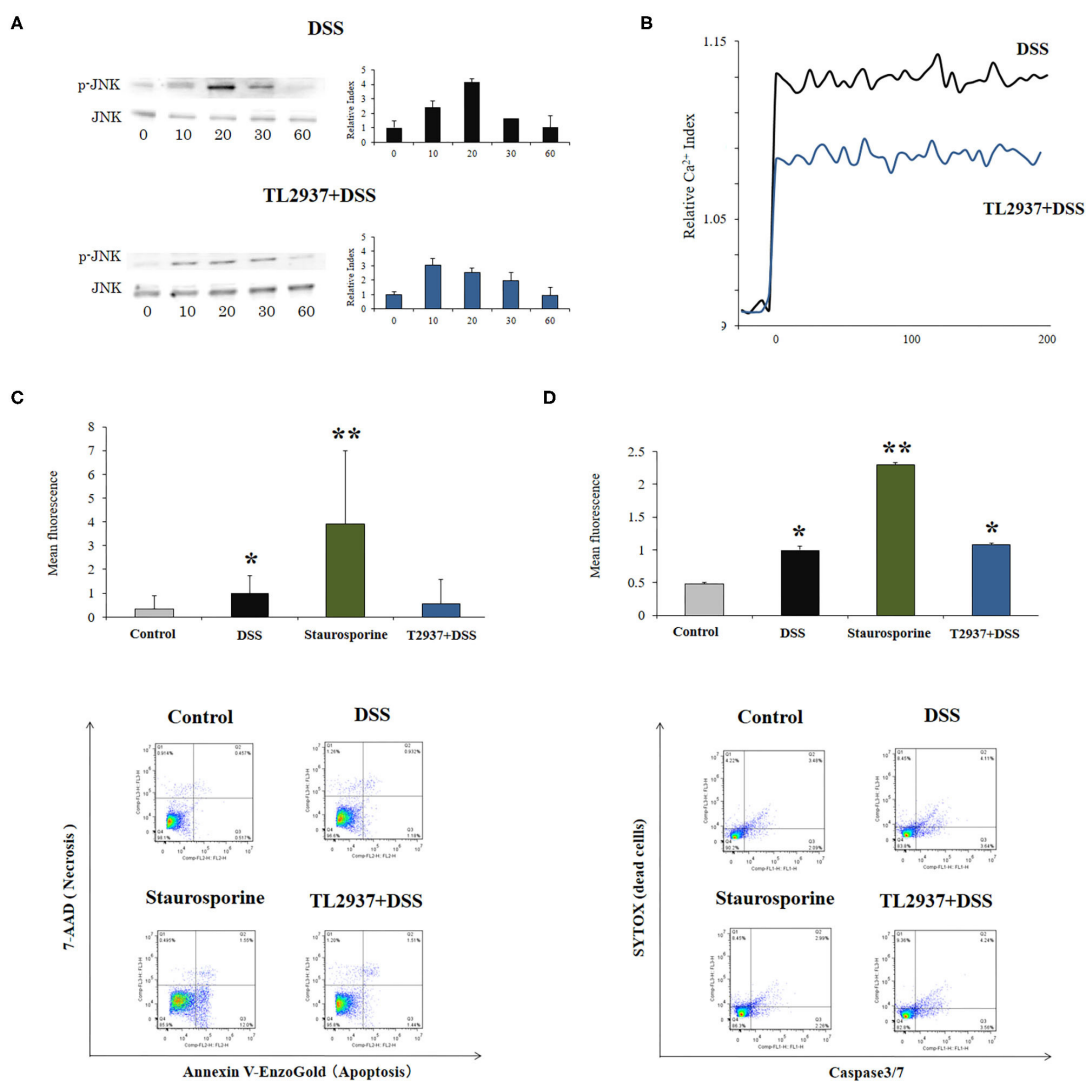
Effect of immunobiotic *Lactobacillus jensenii* TL2937 on the *in vitro* epithelial barrier alterations induced by dextran sodium sulfate (DSS) administration. Porcine intestinal epithelia (PIE) were stimulated with *L. jensenii* TL2937 (5 × 10^7^ cells/ml) for 48 h and then challenged with 0.01% DSS for 6 h. PIE cells challenged only with DSS were used as controls. **(A)** JNK and phosphorylated JNK (p-JNK) levels, **(B)** Ca^+2^ influx, **(C)** apoptosis, and **(D)** caspase 3/7 expression were determined in PIE cells after the challenge with DSS. The results represent three independent experiments. Significant differences when compared to the unchallenged control group: ^*^P < 0.05, ^**^P < 0.01.

The C-Jun N-terminal kinase (JNK) is an intracellular signaling factor that has been reported to be activated in the intestine of human IBD patients (37, 38). In particular, the intracellular epithelial signaling mediated by Ca^2+^-Ask1-MKK7-JNK2-c-Src cascade is involved in DSS-induced tight-junction breakdown (39). Then, the activation of JNK (Figure 6A) and intracellular Ca^2+^ mobilization (Figure 6B) were evaluated in PIE cells challenged with DSS. It was observed that the phosphorylated JNK (p-JNK) protein was increased in control PIE cells between 10 and 20 min (relative index of 4.1 in the peak) after DSS challenge (Figure 6A). The p-JNK returned to basal levels after 30 min in control DSS-challenged PIE cells. In addition, DSS stimulation induced a significant increase in intracellular Ca^2+^ fluxes in PIE cells (Figure 6B). An earlier and lower increase (relative index of 3.0 in the peak) in p-JNK levels (Figure 6A) as well as lower intracellular Ca^2+^ fluxes (Figure 6B) were detected in TL2937-treated PIE after DSS challenge when compared to controls.

The phosphorylation of JNK in the intestinal epithelium has been linked not only to the reduction of tight junctions strength (39), but in addition to the expression of pro-inflammatory cytokines such as IL-6 and TNF-α (40) and the induction of apoptosis (41). Therefore, we also evaluated the effect of DSS in the induction of apoptosis in PIE cells. We performed flow cytometry studies using Annexin V as a probe for phosphatidylserine on the outer membrane of apoptotic cells (Figure 6C) as well as caspase 3/7 (Figure 6D). It was observed that DSS administration significantly increased apoptotic cells when compared to unchallenged controls. However, the apoptosis induced by DSS was lower than that found in PIE cells treated with the well-known apoptosis inductor staurosporine (Figures 6C,D). Of note, the induction of apoptotic cells by DSS in TL2937-treated PIE cells was significantly lower than that of DSS controls and resembled that found in unchallenged PIE cells when Annexin V was used (Figure 6C). However, no differences were found between control and TL2937-treated PIE cells after the challenge with DSS when caspase 3/7 was evaluated (Figure 6D).

### *L. jensenii* TL2937 Differentially Regulates DSS-Triggered Transcriptomic Response in Epithelial Cells

The transcriptomic response of PIE cells to the challenge with DSS and the effect of *L. jensenii* TL2937 in that response were evaluated by microarray analysis. When DSS-treated PIE cells were compared with unchallenged cells, it was found that there were 243 and 327 unique transcripts upregulated and downregulated, respectively (Supplementary Figure 1A). Out of these differentially regulated genes, several were assigned to immune-related functions according to the PPI network (Supplementary Figure 1B) and GO database (Supplementary Figure 1C) analysis. The PPI network is a hierarchical structure, where the Hubs show the factors that play a central role in directing cellular response to a given stimulus. Then, to identify the regulatory Hub genes involved in the transcriptome network of TL2937-treated PIE cells after the challenge with DSS, we constructed and visualized the PPI network of differentially expressed transcripts (Supplementary Figure 1B). The centrality measures (degree and betweenness) indicated that *UBC, UBQLN4, PTN, ALB, IL2, ONECUT1, VTN, TTR, IFR5, APP, NUP214, RARB, FGFR2, TF, FOXO4, AMBP, DAB2, GRB2, CFH*, and *SMAD3* were the major Hub genes of the *L. jensenii* TL2937-mediated transcriptional network in the DSS-treated PIE cells (Supplementary Figure 1B; Supplementary Table 3). The changes of immunotranscriptome response in PIE cells after DSS challenge included genes in the following GO Biological Process pathways: “Response to stimulus,” “Regulation of response to stimulus,” “Cell surface receptor signaling pathway,” “response to stress,” “Defense response,” “Regulation of immune system process,” “Regulation of cytokine production,” “Regulation of cell adhesion,” and “Regulation of MAPK cascade” (Supplementary Figure 1C).

**Figure 7:**
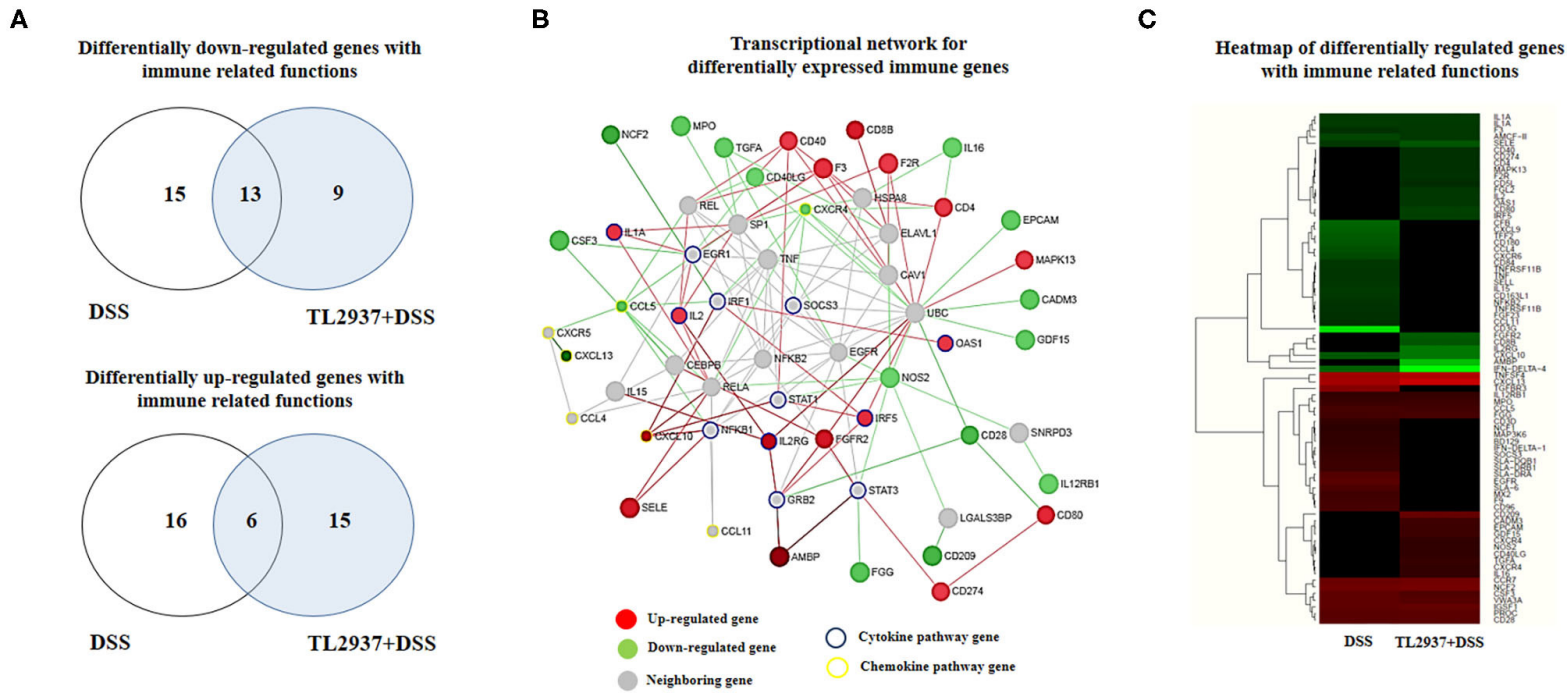
Effect of immunobiotic *Lactobacillus jensenii* TL2937 on the immunotranscriptomic response of intestinal epithelial cells induced by dextran sodium sulfate (DSS) administration. Porcine intestinal epithelial (PIE) were stimulated with *L. jensenii* TL2937 (5 × 10^7^ cells/ml) for 48 h and then challenged with 0.01% DSS for 6 h. PIE cells challenged only with DSS were used as controls. The expression of differentially regulated genes with immune-related functions was evaluated by microarray analysis. **(A)** Venn diagrams showing the number of differentially upregulated and downregulated genes with immune-related functions for each experimental group. **(B)** PPI network and **(C)** heat-map analysis of genes with immune-related functions.

Immune-related genes were found in both upregulated (37 unique genes) and downregulated (37 unique genes) transcript groups (Figure 7A). In order to identify the major immunoregulatory Hub genes involved in the transcriptomic network of TL2937-treated PIE cells after the challenge with DSS, we constructed and visualized the PPI network of differentially expressed immune related genes (Figure 7B). The network displayed that *MAPK13, CD80, CD274, CD8B, CD40, CD4, F2R, F3, OAS1, IRF5, FGFR2, IL2RG, AMBP, IL2, IL1A, SELE*, and *CXCL10* were upregulated among the regulatory Hubs. While *NOS2, IL16, EPCAM, CADM3, GDF15, IL12RB1, CD28, CD209, FGG, CXCR4, TGFA, MPO, NCF2, CSF3, CCL5*, and *CXCL13* were downregulated in PIE cells among the regulatory Hub genes of the immunotranscriptomic networks (Figure 7B). Significant differences were observed in the immunotranscriptomic responses when TL2937-treated PIE cells were compared with DSS controls (Figure 7C; Supplementary Table 4). The most remarkable changes in TL2937-treated PIE cells after stimulation with DSS were found in expression cytokines, chemokines, and adhesion molecules.

### *L. jensenii* TL2937 Differentially Regulates the DSS-Triggered Inflammatory Response in Epithelial Cells

**Figure 8:**
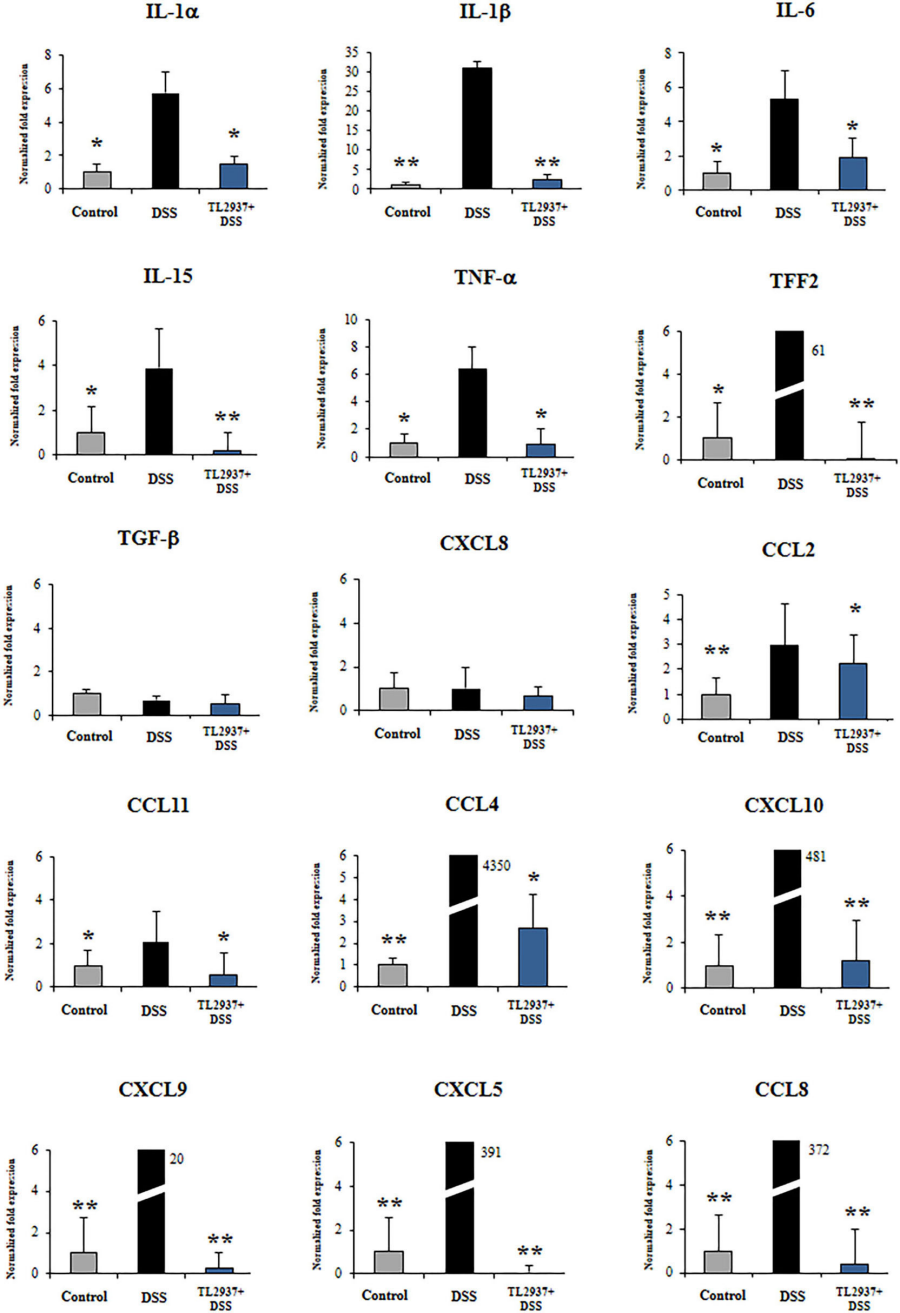
Effect of immunobiotic *Lactobacillus jensenii* TL2937 on the immunotranscriptomic response of intestinal epithelial cells induced by dextran sodium sulfate (DSS) administration. Porcine intestinal epithelia (PIE) were stimulated with *L. jensenii* TL2937 (5 × 10^7^ cells/ml) for 48 h and then challenged with 0.01% DSS for 6 h. PIE cells challenged only with DSS were used as controls. The expression levels of *TNF*-α, *IL-1*α, *IL-1*β, *IL-6, IL-15, TFF2, TGF*-β, *CCL2, CCL4, CCL8, CCL11, CXCL5, CXCL8, CXCL9*, and *CXCL10* were determined by qRT-PCR. The results represent three independent experiments. Significant differences when compared to the DSS control group: ^*^P < 0.05, ^**^P < 0.01.

**Figure 9:**
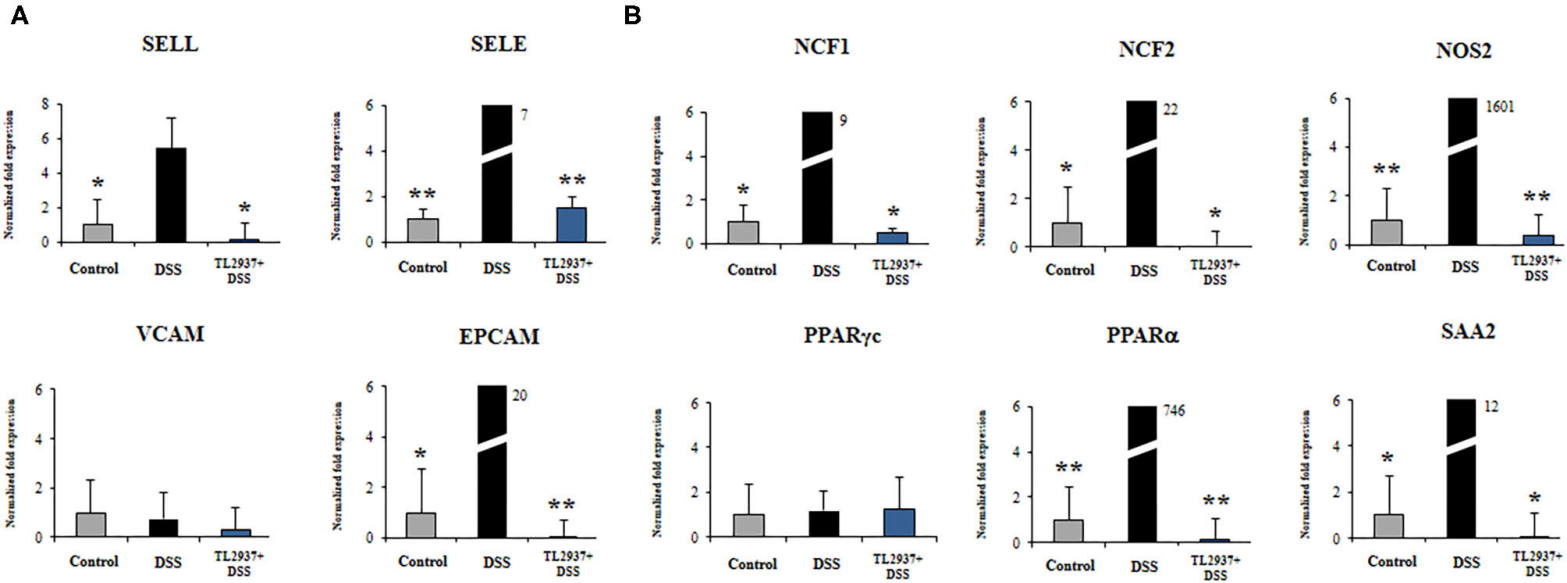
Effect of immunobiotic Lactobacillus jensenii TL2937 on the immunotranscriptomic response of intestinal epithelial cells induced by dextran sodium sulfate (DSS) administration. Porcine intestinal epithelial (PIE) were stimulated with L. jensenii TL2937 (5 × 10^7^ cells/ml) for 48 h, and then challenged with 0.01% DSS for 6 h. PIE cells challenged only with DSS were used as controls. The expression levels of adhesion molecules **(A)** *SELE, SELL, VCAM, EPCAM*, and inflammatory factors **(B)** *NCF1, NCF2, NOS2, c, PPAR*α, and SAA2 were determined by qRT-PCR. The results PPAR represent three independent experiments. Significant differences when compared to the DSS control group: ^*^P < 0.05, ^**^P < 0.01.

To further evaluate gene expression changes induced by DSS in PIE cells, qRT-PCR was performed. From the immune and immune-related genes differentially regulated by DSS in the microarray analysis, we selected 25 belonging to cytokines, chemokines (Figure 8), and adhesion molecules (Figure 9A) groups as well as *NCF1, NCF2, NOS2, PPAR*γ*c, PPAR*α, and *SAA2* (Figure 9B) to be studied by qRT-PCR.

The challenge of PIE cell with DSS significantly increased the expression of *TNF*-α, *IL-1*α, *IL-1*β, *IL-6, IL-15*, and *TFF2* when compared to unchallenged cells (Figure 8). DSS stimulation also induced a remarkable increase in the expression of *CCL4, CCL8, CXCL5, CXCL9*, and *CXCL10* as well as modest but significant increases in *CCL2* and *CCL11* (Figure 8). Of note, TL2937-treated PIE cells showed expression values of these cytokines and chemokines (Figure 8) that were lower than the DSS control cells and were similar to those found in unchallenged PIE cells. In our hands, no significant variations in the expression of *TGF*-β and *CXCL8* were found when the different experimental groups were compared to unchallenged PIE cells.

As shown in Figure 9A, significant higher expression levels of *SELL, SELE*, and *EPCAM* were found when PIE cells stimulated with DSS were compared to unchallenged cells. The expression levels of those adhesion molecules in TL2937-treated PIE cells were lower than the DSS controls and were similar to those observed in unchallenged PIE cells. No significant variations in the expression of *VCAM* were found when the different experimental groups were compared to unchallenged PIE cells.

DSS stimulation induced increases in the expression levels of *NCF1, NCF2, NOS2, PPAR*α, and *SAA2* (Figure 9B). Lower expression levels of these factors were found in TL2937-treated PIE cells when compared the DSS control cells. Moreover, the expression levels were similar to those found in unchallenged PIE cells (Figure 9B). No significant variations in the expression of *PPAR*γ*c* were found when the different experimental groups were compared to unchallenged PIE cells (Figure 9B).

**Figure 10:**
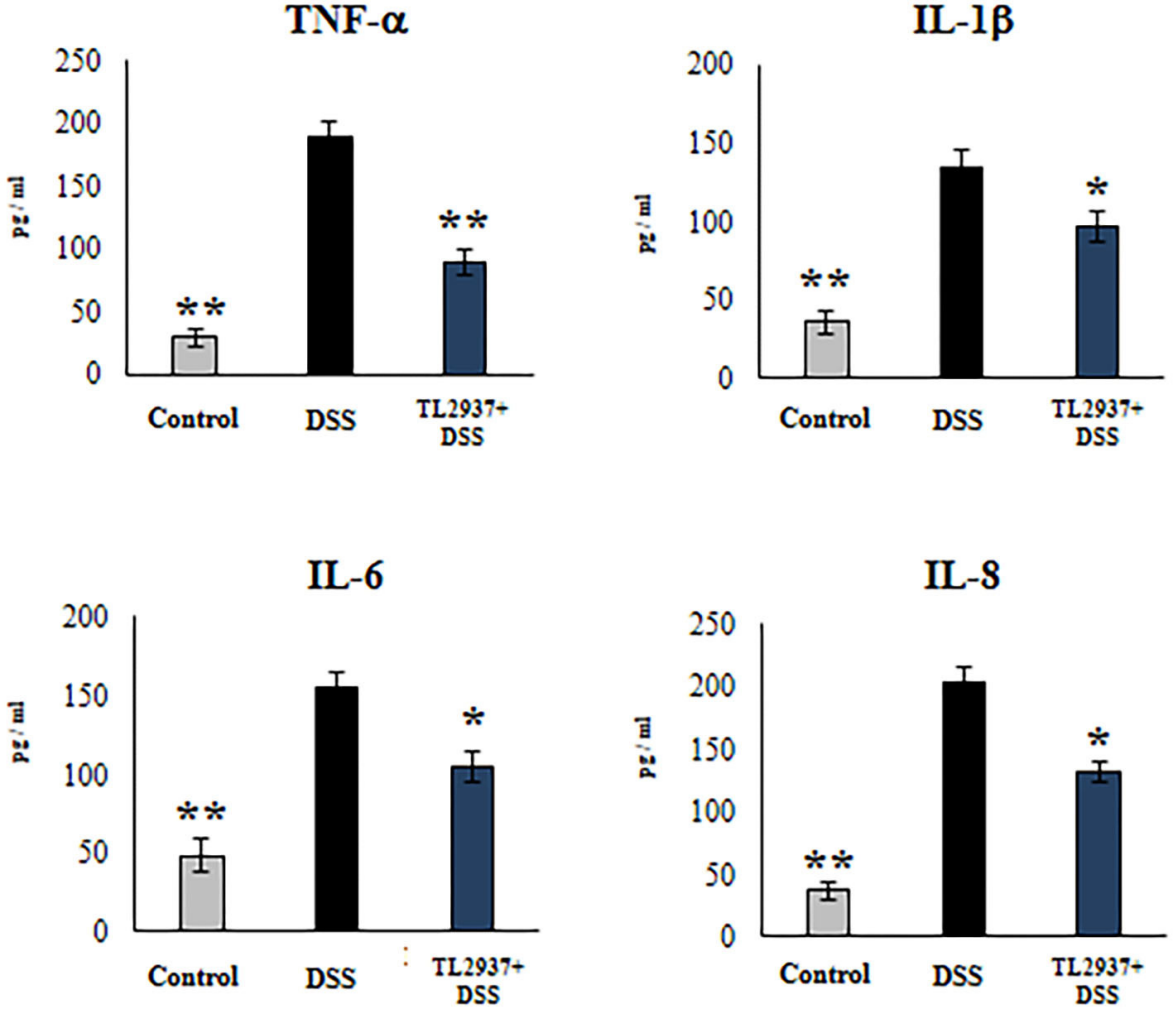
Effect of immunobiotic *Lactobacillus jensenii* TL2937 on the inflammatory cytokine production by human colon Caco-2 cells induced by dextran sodium sulfate (DSS) administration. Caco-2 cells were stimulated with *L. jensenii* TL2937 (5 × 10^7^ cells/ml) for 48 h and then challenged with 1% DSS for 48 h. Caco-2 cells challenged only with DSS were used as controls. The levels of TNF-α, IL-1β, IL-6, and IL-8 were determined by ELISA. The results represent three independent experiments. Significant differences when compared to the DSS control group: ^*^P < 0.05, ^**^P < 0.01.

In order to confirm the immunomodulatory effect of the TL2937 strain on IECs and validate our PIE cell system as a potential human model, we performed experiments in human Caco-2 cells (29). For this purpose, Caco-2 cells were treated with *L. jensenii* TL2937, challenged with 1% DSS, and the levels of TNF-α, IL-1β, IL-6, and IL-8 were measured in culture supernatants (Figure 10). The challenge of Caco-2 cells with DSS significantly increased the levels of the four cytokines when compared to unchallenged cells. Of note, the levels of TNF-α, IL-1β, IL-6, and IL-8 were significantly reduced in lactobacilli-treated cells when compared to DSS controls.

## Discussion

The regulation of the cytokine networks that control the intestinal epithelial barrier function and the mucosal immune system has emerged as an interesting alternative for IBD treatment (1, 2, 4). Moreover, the great progress in the understanding of how beneficial microbes are able to modulate mucosal cytokines has allowed the selection and characterization of immunobiotic candidates for alleviating intestinal inflammation in IBD patients (12, 13). In this work, by using a DSS-induced colitis in the mouse model, we have demonstrated for the first time the ability of the immunobiotic strain *L. jensenii* TL2937 to protect the intestinal epithelium from the inflammatory damage through the regulation of the intestinal cytokine network.

In our hands, the administration of *L. jensenii* TL2937 before the challenge of mice with DSS achieved the protective effect. In fact, the preventive administration (PRE DSS+TL2937 group) or the one that started before and then was administered simultaneously with DSS (CONT DSS+TL2937 group) showed the ability to improve the general health status of animals and to reduce intestinal disorders mediated by the inflammatory response. Those beneficial effects were not observed when the immunobiotic bacteria were administered only simultaneously with DSS (SIM DSS+TL2937 group). Our results are in line with several other reports that have indicated that immunobiotic preventive treatments are more effective to alleviate the symptoms of colitis than their administration during acute active inflammatory phases [reviewed in (12)]. For example, it was reported that the preventive treatment with *Bifidobacterium breve* NCC2950 significantly diminished the severity of DSS-induced colitis, IEC damage, and MPO activity by the regulation of pro- and anti-inflammatory cytokine balance, while the administration of this strain during active DSS colitis was not effective to control inflammation (42). Similarly, only the preventive administration of TL2937 strain was able to improve body weight gain, diminish DAI and clinical score values, reduce the alterations of colon length and the colon length:weight ratio, and diminish intestinal MPO activity. Moreover, our results evidenced a remarkable capacity of *L. jensenii* TL2937 to differentially regulate the intestinal cytokine profile in DSS-challenged mice.

The concentrations of TNF-α, IL-1β, IL-6, IFN-γ, and IL-17 were significantly lower in the colon of *L. jensenii* TL2937-treated mice than in the DSS control group. All these inflammatory cytokines have been implicated in the pathogenesis of IBD. Earlier studies demonstrated the detrimental effects of TNF-α in IBD and that the blockade of this cytokine was effective in ameliorating the severity of colitis in both the T cell transfer (43) and the DSS mouse models (44). It was also reported that the excessive production of TNF-α weakened the intestinal epithelial barrier function by altering IEC integrity and inducing their apoptosis (45, 46). The interaction of TNF-α with the receptors TNFR1 and TNFR2 stimulates the expression of inflammatory factors through NF-κB activation, induces damage of IECs *via* myosin light chain kinase (MLCK) activation (47), and stimulates cell death through receptor-interacting protein kinase 1 (RIPK1) and caspase protein activation (48). Moreover, a plethora of effects have been attributed to TNF-α during the generation and development of IBD including the induction of death in Paneth cells, the increase in angiogenesis, the enhancement of matrix metalloproteinase production, and the potentiation of inflammation by the stimulation of immune cells (48). On the other hand, although IL-6 is of importance for the maintenance of the normal barrier function of the intestinal epithelium (49), deregulated production of IL-6 in the gut promotes T cell expansion and stimulates Th1 cell-mediated inflammation (50). It was also demonstrated that the deletion of the inflammasome component caspase-1 that is involved in the production of the active forms of IL-1β and IL-18 (51) or the blockade of IL-1β or IL-18 signaling (52, 53) significantly attenuates the severity of DSS-induced colitis in mice. In addition, IL-1β is able to promote intestinal inflammatory damage by collaborating in the activation and differentiation of T cells that produce IL-17 or IFN-γ (54), which are increased and are highly active in the intestinal mucosa of IBD patients (1, 2). On the other hand, it was found that IL-10 and IL-27 were significantly higher in *L. jensenii* TL2937-treated mice than in the DSS control group while no differences between the groups were observed for colon TGF-β concentrations. It was shown that mice deficient in either IL-10 or its receptor IL-10R develop spontaneous colitis (55, 56). Furthermore, genetic studies evaluating IL-10 found that some mutations in the genes encoding IL-10 or IL-10R are associated with an early onset of IBD development in children (57) while deficiencies in IL-10 have been associated with pathogenic responses of IL-12- and IL-23-producing T cells in several models of colitis (2). It was also shown that the alterations in both IL-35 and IL-27 exacerbate T cell transfer colitis (58). Then, the results of this work indicate that *L. jensenii* TL2937 is able to induce a differential cytokine balance in response to DSS stimulation by decreasing the production of inflammatory cytokines and increasing the levels of regulatory cytokines. This differential profile could be related to a direct effect of the immunobiotic strain on cytokine production, as observed on day −4 for IL-10 and IL-27, as well as an indirect effect due to the protection of the intestinal epithelium against the damage that decreases the activation of the innate immune response.

The murine model of DSS-induced colitis is particularly useful for the evaluation of the role of both intestinal barrier and the innate immune responses in the context of IBD (12). In this mouse model, the administration of DSS in the drinking water induces damage on IECs causing alterations in the barrier functions of the surface epithelium and allowing the passage of lumen antigens that strongly stimulate the innate immune system. Then, considering the *in vivo* studies performed here demonstrating the ability of *L. jensenii* TL2937 to protect mice against DSS challenge, we hypothesized that the immunobiotic strain would exert its beneficial effects, at least partially, through the modulation of IEC physiology. In fact, our experiments evaluating the expression of tight junction and adherens junction genes *in vivo*, including *Ocln, ZO-1, Cldn1*, and *Ctnn*, demonstrated the ability of *L. jensenii* TL2937 to strengthen the barrier function of IECs. Then, detailed *in vitro* studies were performed by using the PIE cell line and DSS stimulation.

**Figure 11:**
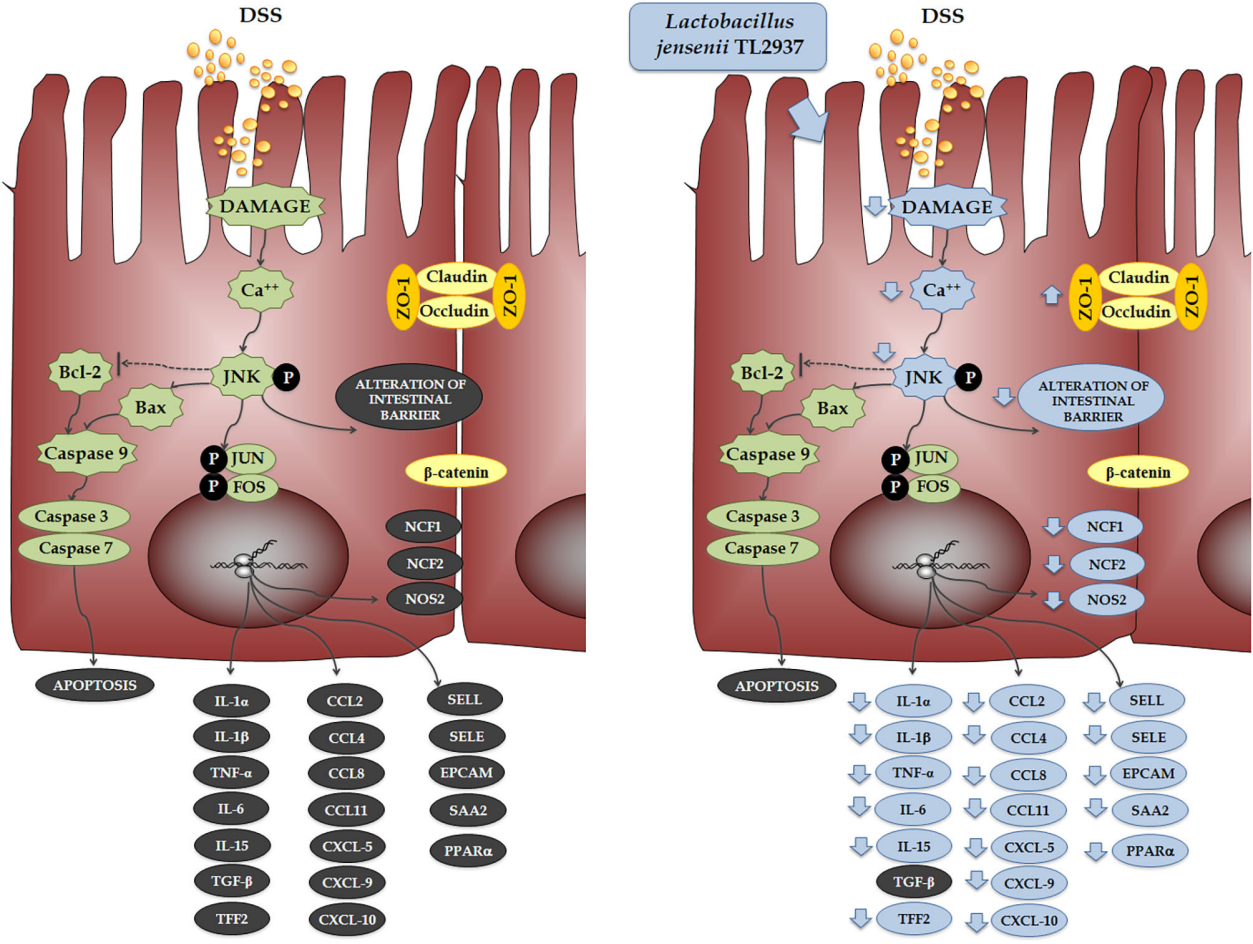
Effect of immunobiotic *Lactobacillus jensenii* TL2937 on the immunotranscriptomic response of intestinal epithelial cells induced by dextran sodium sulfate (DSS) administration. Global overview of the signaling pathways and immune genes differentially regulated in porcine intestinal epithelial (PIE) cells after the challenge with DSS. Global overview of the signaling pathways and immune genes differentially regulated in PIE cells treated with *L. jensenii* TL2937 and challenged with DSS.

When the effect of *L. jensenii* TL2937 on the immunotranscriptome response of DSS-challenged PIE cells was evaluated, the most remarkable differences were found in the expression of inflammatory cytokines and chemokines (Figure 11). Significantly reduced expression levels of *TNF*-α*, IL-1*α, *IL-1*β, *IL-6*, and *IL-15* were found in DSS-challenged PIE cells previously stimulated with the TL2937 strain, which showed a strong correlation with our *in vivo* findings in mice and with our studies in human Caco-2 cells. In addition to inflammatory cytokines, IECs are able to secrete chemokines and express adhesion molecules in response to injury, and in this way, these cells play an active role in shaping the nature of the intestinal immune response. It was shown that the chronic intestinal inflammation of IBD is driven and sustained in part by an increased production of chemokines from the inflamed epithelium (59). In this work, the challenge of PIE cells with DSS significantly increased their expression of *CCL2, CCL4, CCL8, CXCL5, CXCL9*, and *CXCL10* as well as the adhesion molecules *SELL, SELE*, and *EPCAM*, highlighting the role of inflamed IECs in the recruitment and activation of leukocytes in the intestinal mucosa. Of note, most of the chemokines and adhesion molecules evaluated were lower in TL2937-treated PIE cells when compared to DSS control. Although we have not performed a detailed evaluation of chemokines and adhesion molecules in the DSS mouse model, we observed a significant reduction in the levels of CXCL1, MCP-1, IL-15, and MPO in the colon of TL2937-treated mice. It is tempting to speculate that *L. jensenii* TL2937 would be able to beneficially modulate chemokines and adhesion molecules *in vivo*. It would be of value to evaluate the effect of the immunobiotic TL2937 strain on the recruitment and activation of leukocytes in the intestinal mucosa, and the connection of this effect to the protection against the intestinal damage. This could lay the scientific basis for the application of this strain in other inflammatory-based pathologies.

The cytokines produced by IECs and cells in the surrounding microenvironment are key players in the control of the migration, differentiation, and survival of IECs. The closure of erosions and ulcers in the intestinal mucosa is promoted by the proliferation and expansion of IECs and is critically dependent on intestinal cytokine networks (60, 61). Studies have found that increased apoptosis of IECs in the inflamed mucosa of IBD patients and the overproduction of inflammatory cytokines have been associated with the destruction of intestinal epithelial layers by promoting apoptotic death in IECs (62). Moreover, some inflammatory cytokines such as TNF-α and IFN-γ are able to alter the molecular bonds between the IECs reducing the epithelial barrier function and aggravating the epithelial erosions (47, 63). Here, we were able to reproduce *in vitro* those relevant characteristics of the intestinal mucosa of IBD patients. In our hands, DSS administration to PIE cells significantly increased the numbers of apoptotic cells and diminished the TER index. Moreover, in line with our results in mice challenged with DSS, PIE cells had an altered expression and distribution of ZO-1 and β-catenin, indicating a clear alteration of the epithelial barrier. Interestingly, *L. jensenii* TL2937 was able to significantly reduce the alterations of all these parameters in DSS-challenged PIE cells. As mentioned earlier, the phosphorylation of JNK in the intestinal epithelium has been linked to some cellular and molecular changes that are characteristic of IBD. JNK pathway activation is related the expression of TNF-α (40), the reduction of tight junctions strength (39), and the induction of apoptosis (41). In our *in vitro* model, a clear activation of the JNK pathway was observed in PIE cells after the challenge with DSS. Moreover, we found that *L. jensenii* TL2937 was able to differentially modulate the activation of this pathway, since an earlier and lower increase in p-JNK levels and lower intracellular Ca^2+^ fluxes were detected in this group. Previously, we evaluated the interaction of *L. jensenii* TL2937 with PIE cells and demonstrated that this bacterium is able to interact with TLR2 and upregulate the expression of negative regulators of the TLR signaling pathway (17). In this way, this immunobiotic strain modulates the subsequent TLR4 activation by reducing the activation of NF-κB and p38 signaling pathways, and the expression of inflammatory cytokines and chemokines. It is tempting to speculate that in the context of DSS stimulation, the upregulation of negative regulators induced by the TL2937 strain would allow the regulation of the JNK pathway and significantly impact in the immunotranscriptomic response, apoptosis, and barrier functions of PIE cells.

Because of the anatomical, physiological, and immunological similarities of human and porcine intestine, pigs have been considered an attractive model to study the mechanisms involved in intestinal diseases as well as the interaction of microbes with the immune system (64). In this work, we demonstrated that PIE cells could give us the possibility of conducting *in vitro* studies to evaluate the epithelial barrier function, the innate immune response, and the influence of immunomodulatory beneficial microbes on those parameters, in the context of DSS challenge. Our statement is supported by the good correlation found in the modulation of cytokines and chemokines by *L. jensenii* TL2937 in the *in vivo* mouse model and the *in vitro* PIE cell system. Moreover, we also found that the TL2937 strain was capable of reducing the production of inflammatory cytokines and chemokines in human Caco-2 cells in response to DSS, resembling the results observed in PIE cells. Caco-2 monolayers have been successfully used for the evaluation of therapeutic alternatives on the barrier integrity, inflammation, and cell death in response to DSS (29, 65–67). Then, our comparative *in vitro* results reinforce the scientific basis for proposing PIE cells as a good *in vitro* model for molecular studies of IBD. Further comparative studies that involve the use of lactobacilli that do not have beneficial effects on the inflammatory response and/or the epithelial barrier in IBD models would be of great importance to position our *in vitro* system as a useful tool in the selection of potential probiotics for UC or CD.

## Conclusions

The findings of this work allow us to arrive at three main conclusions: (a) *L. jensenii* TL2937 is able to alleviate DSS-induced colitis through the regulation of intestinal cytokine networks suggesting a potential novel application for this immunobiotic strain in the context of IBD. Furthermore, (b) the modulation of the transcriptomic response of IECs by the TL2937 strain would play a key role in this beneficial effect. Although a remarkable effect of *L. jensenii* TL2937 on IECs was observed here, further studies are needed to characterize the mechanisms involved in the protection against DSS colitis induced by this immunobiotic strain. For example, it is known that IECs are not efficient producers of IL-10 or IL-27, two cytokines that were augmented in mice treated with *L. jensenii* TL2937. Then, the study of the cellular and molecular interactions of the TL2937 strain with intestinal macrophages or DCs would be of value to explain the increases of the regulatory cytokines. In addition, we demonstrated that (c) the *in vitro* PIE cell immunoassay system could be of value for the screening and selection of new immunobiotic strains for their application in IBD. Although murine models have been of great value to select and characterize immunobiotics for IBD, the reduction of animals being used in experiments has become a central topic of debate in the scientific community (68). The good correlation between the alterations of barrier function, epithelial cell death, and cytokine networks found in previous *in vitro* and *in vivo* IBD models and our *in vitro* findings in PIE cells show the potential value of our system to select efficiently new immunobiotic candidates for CD or UC.

## Data Availability Statement

The datasets presented in this study can be found in online repositories. The names of the repository/repositories and accession number(s) can be found in the article/Supplementary Material.

## Ethics Statement

The animal study was reviewed and approved by Ethics Committee for Animal Experiments CERELA-CONICET.

## Author Contributions

JV and HK designed the study and manuscript writing. NS, VG-C, MY, and MT did the laboratory work. MI and LA did the microarray and statistical analysis. AG-C, WI-O, HT, JV, and HK contributed the data analysis and interpretation. JV, HT, and HK provided the resources. All authors read and approved the manuscript.

## Conflict of Interest

The authors declare that the research was conducted in the absence of any commercial or financial relationships that could be construed as a potential conflict of interest.

## Supplementary Material

Supplementary Figure 1 Effect of immunobiotic *Lactobacillus jensenii* TL2937 on the transcriptomic response of intestinal epithelial cells induced by dextran sodium sulfate (DSS) administration. Porcine intestinal epithelial (PIE) were stimulated with *L. jensenii* TL2937 (5 × 10^7^ cells/ml) for 48 h, and then challenged with 0.01% DSS for 6 h. PIE cells challenged only with DSS were used as controls. The expression of differentially regulated genes was evaluated by microarray analysis. **(A)** Venn diagrams showing the number of differentially upregulated and downregulated genes for each experimental group. **(B)** PPI network of differentially regulated genes. **(C)** Number of matched genes categorized according to Gene Ontology database.

Supplementary Table 1 Disease activity index.

Supplementary Table 2 Primer used for two-step real-time quantitative PCR (qRT-PCR).

Supplementary Table 3 Transcriptional network for differentially expressed genes.

Supplementary Table 4 List of differentially expressed genes induced by immunobiotic Lactobacillus jensenii TL2937 in porcine intestinal epithelial (PIE) stimulated with dextran sodium sulfate (DSS).
