# Supplementary figures and images for: Immunobiotic Lactobacillus jensenii TL2937 Alleviates Dextran Sodium Sulfate-Induced Colitis by Differentially Modulating the Transcriptomic Response of Intestinal Epithelial Cells
Source: Front Immunol. 2020 Sep 17;11:2174. doi: 10.3389/fimmu.2020.02174 (PMC7527445; doi:10.3389/fimmu.2020.02174)

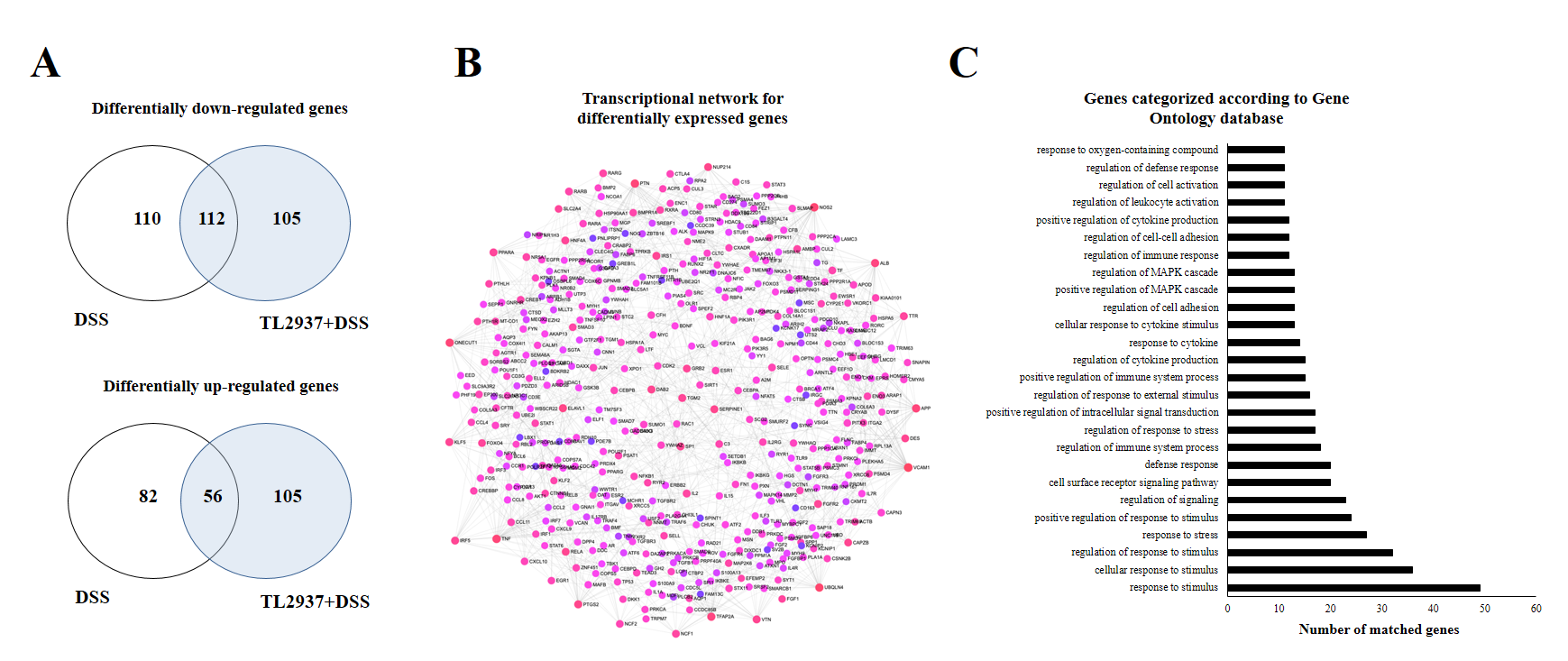

Supplement: Supplementary Figure 1 — Effect of immunobiotic Lactobacillus jensenii TL2937 on the transcriptomic response of intestinal epithelial cells induced by dextran sodium sulfate (DSS) administration. Porcine intestinal epithelial (PIE) were stimulated with L. jensenii TL2937 (5 × 10^7^ cells/ml) for 48 h, and then challenged with 0.01% DSS for 6 h. PIE cells challenged only with DSS were used as controls. The expression of differentially regulated genes was evaluated by microarray analysis. (A) Venn diagrams showing the number of differentially upregulated and downregulated genes for each experimental group. (B) PPI network of differentially regulated genes. (C) Number of matched genes categorized according to Gene Ontology database. [file Image_1.TIF]
