# Supplementary material for: Immunobiotic Lactobacillus jensenii TL2937 Alleviates Dextran Sodium Sulfate-Induced Colitis by Differentially Modulating the Transcriptomic Response of Intestinal Epithelial Cells
Source: Front Immunol. 2020 Sep 17;11:2174. doi: 10.3389/fimmu.2020.02174 (PMC7527445; doi:10.3389/fimmu.2020.02174)
Supplement: Supplementary Table 1 — Disease activity index. [file Table_1.docx]

**Supplementary Table 1.** Disease activity index.

| **Weight loss** | **Feces** | **Blood** | **General appearance** |
| --- | --- | --- | --- |
| No change = 0 | Normal = 0 | No blood = 0 | Normal = 0 |
| Below 5% = 1 | Pasty or semiformed = 2 | Visible blood in rectum = 1 | Piloerection = 1 |
| Between 6 and 10% = 2 | Liquid and sticky = 4 | Visible blood on fur = 2 | Lethargy and piloerection = 2 |
| Between 11 and 20% = 3 |  |  | Motionless and sickly = 4 |
| Above 20% = 4 |  |  |  |

The disease activity index was calculated considering the weight loss percentage, the presence of fecal blood, and stool consistency. DAI values were calculated as [(weight loss score) + (stool consistency) + (rectal bleeding score)]/4 and scored on a 0±4 scale according to (23) and (24).
