# Supplementary material for: Immunobiotic Lactobacillus jensenii TL2937 Alleviates Dextran Sodium Sulfate-Induced Colitis by Differentially Modulating the Transcriptomic Response of Intestinal Epithelial Cells
Source: Front Immunol. 2020 Sep 17;11:2174. doi: 10.3389/fimmu.2020.02174 (PMC7527445; doi:10.3389/fimmu.2020.02174)
Supplement: Supplementary Table 2 — Primer used for two-step real-time quantitative PCR (qRT-PCR). [file Table_2.docx]

**Supplementary Table 2.** Primer used for two-step real-time quantitative PCR (qRT-PCR).

| **Gene** | **Host** | **Forward primer (5'-3')** | **Reverse primer (5'-3')** |
| --- | --- | --- | --- |
| *NCF1* | Pig | TTCATAAGCTCCTGGATGGC | GCTCTTGATCTTGCTTTGGG |
| *NCF2* | Pig | TGTCCTGAAGAAGTGCAACG | GGCTGTTGCTGAGAATGGAT |
| *PPARγC1* | Pig | TAAAGATGCCGCCTCTGACT | TGACCGAAGTGCTTGTTCAG |
| *TFF2* | Pig | GGGGAGGTTATCTTGCTTGC | GCAGTTGACCCGGTTCTTG |
| *SELL* | Pig | GTGATGCAGGGTACTACGGG | AGAACTTGCCCAAAGGGTGA |
| *EPCAM* | Pig | GCGATAGCGATTGTTGCTGG | CCCTATGCATCTCGCCCATC |
| *SELE* | Pig | ACCCCAGAGGCCACCAA | CCTCGTTGCTTTGCTTATTATTTG |
| *VCAM* | Pig | CTGCTCTAGTGATGGCTTTCCA | CCATCCCTAAGCTTCTTGCT |
| *NOS2* | Pig | CAGCCAGGTGCTCACCTATT | TCTCAAGCCTCTGCCTTTCG |
| *SAA2* | Pig | AGAGCCTACRCGGACATGAGAGA | CCCCGGGCATGGAAGTAC |
| *PPARα* | Pig | CGACCTGGAAAGCCCGTTAT | GGATCCATCTGATCCCGGAC |
| *IL-1β* | Pig | GCCCTGATCCCCAACTGG TA | CCAGGAAGACGGGCTTTTG |
| *IL-6* | Pig | TGGATAAGCTGCAGTCACAG | ATTATCCGAATGGCCCTCAG |
| *IL-8* | Pig | GCTCTCTGTGAGGCTGCAGTT | TTTATGCACTGGCATCGAAGTT |
| *TNFα* | Pig | CGACTCAGTGCCGAGATCAA | CCTGCCCAGATTCAAAG |
| *CCL2* | Pig | ACAGAAGAGTCACCAGCAGCAA | GCCCGCGATGGTCTTG |
| *CXCL10* | Pig | ACCTGTTAATCCGAGGTCCTTAGA | CAACATGTGGGCAAGATTGACT |
| *CCL11* | Pig | CCAAAGAGTCACTGCCAACA | ACTCCATGGCATTCTGAACC |
| *β-actin* | Mouse | GGCTGTATTCCCCTCCATCG | CCAGTTGGTAACAATGCCATGT |
| *Occludin* | Mouse | TTGAAAGTCCACCTCCTTACAGA | CCGGATAAAAAGAGTACGCTGG |
| *ZO-1* | Mouse | GCCGCTAAGAGCACAGCAA | GCCCTCCTTTTAACACATCAGA |
| *Claudin-1* | Mouse | AGACCTGGATTTGCATCTTGGTG | TGCAACATAGGCAGGACAAGAGTTA |
| *β-catenin* | Mouse | ATGACTCGAGCTCAGAGGGT | ATTGCACGTGTGGCAAGTTC |
