# Supplementary material for: Immunobiotic Lactobacillus jensenii TL2937 Alleviates Dextran Sodium Sulfate-Induced Colitis by Differentially Modulating the Transcriptomic Response of Intestinal Epithelial Cells
Source: Front Immunol. 2020 Sep 17;11:2174. doi: 10.3389/fimmu.2020.02174 (PMC7527445; doi:10.3389/fimmu.2020.02174)
Supplement: Supplementary Table 3 — Transcriptional network for differentially expressed genes. [file Table_3.docx]

**Supplementary Table 3.** Transcriptional network for differentially expressed genes.

| **Id** | **Label** | **Degree** | **Betweenness** |
| --- | --- | --- | --- |
| P0CG48 | UBC | 37 | 5925.89 |
| Q9NRR5 | UBQLN4 | 18 | 1810.38 |
| P21246 | PTN | 18 | 1560.47 |
| P02768 | ALB | 16 | 1465.73 |
| P60568 | IL2 | 16 | 1416.98 |
| Q9UBC0 | ONECUT1 | 14 | 1071.9 |
| P04004 | VTN | 14 | 903.6 |
| P02766 | TTR | 14 | 839.67 |
| Q13568 | IRF5 | 13 | 1279.3 |
| P05067 | APP | 13 | 1140.34 |
| P35658 | NUP214 | 12 | 832.03 |
| P10826 | RARB | 12 | 641.67 |
| P21802 | FGFR2 | 11 | 852 |
| P02787 | TF | 11 | 532.05 |
| P98177 | FOXO4 | 10 | 632.55 |
| P02760 | AMBP | 10 | 464.75 |
| P98082 | DAB2 | 9 | 949.45 |
| P62993 | GRB2 | 9 | 574.01 |
| P08603 | CFH | 9 | 459.73 |
| P84022 | SMAD3 | 7 | 456.15 |
| P02788 | LTF | 7 | 446.01 |
| P41235 | HNF4A | 7 | 268.93 |
| P20823 | HNF1A | 7 | 267.3 |
| P35222 | CTNNB1 | 6 | 517.16 |
| Q9Y4E5 | ZNF451 | 6 | 427.63 |
| P12883 | MYH7 | 6 | 391.35 |
| P16220 | CREB1 | 6 | 330.72 |
| Q15717 | ELAVL1 | 6 | 310.37 |
| P08047 | SP1 | 6 | 263.74 |
| Q05066 | SRY | 6 | 229.87 |
| O75364 | PITX3 | 6 | 210.68 |
| Q9Y5Q3 | MAFB | 6 | 140.24 |
| P09871 | C1S | 6 | 138.95 |
| P30968 | GNRHR | 6 | 136.98 |
| P36894 | BMPR1A | 5 | 474.36 |
| P31785 | IL2RG | 5 | 400.13 |
| P05181 | CYP2E1 | 5 | 351.11 |
| Q09472 | EP300 | 5 | 318.39 |
| Q15004 | KIAA0101 | 5 | 266.49 |
| P55036 | PSMD4 | 5 | 210.38 |
| P03372 | ESR1 | 5 | 165.87 |
| P02647 | APOA1 | 5 | 157.2 |
| P05090 | APOD | 5 | 107.83 |
| P19793 | RXRA | 4 | 308.26 |
| P60709 | ACTB | 4 | 283.87 |
| P78310 | CXADR | 4 | 278.29 |
| Q05707 | COL14A1 | 4 | 256.86 |
| P40763 | STAT3 | 4 | 242.84 |
| Q03431 | PTH1R | 4 | 238.01 |
| Q92736 | RYR2 | 4 | 206.37 |
| P11473 | VDR | 4 | 189.26 |
| P40933 | IL15 | 4 | 180.56 |
| Q92793 | CREBBP | 4 | 161.95 |
| P42224 | STAT1 | 4 | 150.16 |
| P46379 | BAG6 | 4 | 143.01 |
| P05412 | JUN | 4 | 123.43 |
| P02753 | RBP4 | 4 | 104.15 |
| Q9Y279 | VSIG4 | 4 | 92.18 |
| P49715 | CEBPA | 4 | 91.1 |
| P16871 | IL7R | 4 | 63.52 |
| P12643 | BMP2 | 3 | 207.2 |
| P18146 | EGR1 | 3 | 196.17 |
| Q7Z4S6 | KIF21A | 3 | 171.82 |
| P78380 | OLR1 | 3 | 165.18 |
| Q13547 | HDAC1 | 3 | 144.51 |
| Q16654 | PDK4 | 3 | 133.94 |
| P13569 | CFTR | 3 | 128.89 |
| Q12873 | CHD3 | 3 | 124.03 |
| Q16531 | DDB1 | 3 | 121.72 |
| P22735 | TGM1 | 3 | 117.38 |
| O00472 | ELL2 | 3 | 116.31 |
| O75400 | PRPF40A | 3 | 110.18 |
| P01023 | A2M | 3 | 104.68 |
| P40261 | NNMT | 3 | 98.98 |
| Q14956 | GPNMB | 3 | 95.3 |
| Q15714 | TSC22D1 | 3 | 91.25 |
| P14859 | POU2F1 | 3 | 86.21 |
| P68104 | EEF1A1 | 3 | 82.15 |
| P01106 | MYC | 3 | 79.81 |
| Q15796 | SMAD2 | 3 | 75.36 |
| Q14653 | IRF3 | 3 | 73.35 |
| P01100 | FOS | 3 | 71.95 |
| Q92482 | AQP3 | 3 | 69.82 |
| Q15599 | SLC9A3R2 | 3 | 65 |
| Q13950 | RUNX2 | 3 | 62.73 |
| P17676 | CEBPB | 3 | 52.35 |
| P06732 | CKM | 3 | 50.62 |
| P34931 | HSPA1L | 3 | 42.85 |
| Q03167 | TGFBR3 | 3 | 42.29 |
| O14980 | XPO1 | 3 | 40.86 |
| P10589 | NR2F1 | 3 | 36.78 |
| P01270 | PTH | 3 | 36.4 |
| Q9BQB6 | VKORC1 | 3 | 34.55 |
| Q9UKV0 | HDAC9 | 3 | 34.49 |
| P63104 | YWHAZ | 3 | 33.13 |
| P27348 | YWHAQ | 3 | 21.93 |
| Q13485 | SMAD4 | 3 | 21.23 |
| P13010 | XRCC5 | 2 | 178 |
| Q15038 | DAZAP2 | 2 | 129.31 |
| P10914 | IRF1 | 2 | 122.04 |
| P78537 | BLOC1S1 | 2 | 108.93 |
| Q9NQZ2 | UTP3 | 2 | 105.21 |
| Q12906 | ILF3 | 2 | 99.24 |
| O95295 | SNAPIN | 2 | 78.78 |
| P04181 | OAT | 2 | 78.04 |
| P06756 | ITGAV | 2 | 74.09 |
| Q92729 | PTPRU | 2 | 67.35 |
| P37173 | TGFBR2 | 2 | 67.13 |
| Q00722 | PLCB2 | 2 | 64.12 |
| O43709 | WBSCR22 | 2 | 61.3 |
| O75582 | RPS6KA5 | 2 | 60.65 |
| Q12824 | SMARCB1 | 2 | 58.18 |
| P01137 | TGFB1 | 2 | 53.15 |
| Q92905 | COPS5 | 2 | 52.89 |
| Q14974 | KPNB1 | 2 | 47.5 |
| Q9UN86 | G3BP2 | 2 | 43.27 |
| Q5HYA8 | TMEM67 | 2 | 43.11 |
| P00325 | ADH1B | 2 | 43 |
| P16949 | STMN1 | 2 | 41.29 |
| P18848 | ATF4 | 2 | 40.7 |
| O43765 | SGTA | 2 | 39.96 |
| P08493 | MGP | 2 | 39.93 |
| P01344 | IGF2 | 2 | 38.87 |
| Q13432 | UNC119 | 2 | 36.76 |
| Q15047 | SETDB1 | 2 | 33.6 |
| Q9C093 | SPEF2 | 2 | 31.22 |
| P23771 | GATA3 | 2 | 29.21 |
| P24941 | CDK2 | 2 | 28.1 |
| Q9UKB3 | DNAJC12 | 2 | 27.41 |
| P09038 | FGF2 | 2 | 27.09 |
| P40429 | RPL13A | 2 | 26.95 |
| Q99459 | CDC5L | 2 | 24.52 |
| P49716 | CEBPD | 2 | 20.29 |
| Q15788 | NCOA1 | 2 | 19.37 |
| P07858 | CTSB | 2 | 18.72 |
| O14964 | HGS | 2 | 18.63 |
| P17612 | PRKACA | 2 | 17.84 |
| Q9Y6N6 | LAMC3 | 2 | 16.13 |
| Q92985 | IRF7 | 2 | 16.12 |
| P42568 | MLLT3 | 2 | 15.94 |
| P06733 | ENO1 | 2 | 14.75 |
| Q9NZM3 | ITSN2 | 2 | 14.62 |
| P10275 | AR | 2 | 14.58 |
| Q13617 | CUL2 | 2 | 14.06 |
| Q16891 | IMMT | 2 | 14.05 |
| Q9NRM6 | IL17RB | 2 | 13.96 |
| Q9UM73 | ALK | 2 | 12.94 |
| P01024 | C3 | 2 | 12.82 |
| P10909 | CLU | 2 | 12.22 |
| Q6QNY0 | BLOC1S3 | 2 | 11.71 |
| P08263 | GSTA1 | 2 | 9.8 |
| P61956 | SUMO2 | 2 | 9.28 |
| P15090 | FABP4 | 2 | 9.26 |
| P17275 | JUNB | 2 | 7.85 |
| P40879 | SLC26A3 | 2 | 7.66 |
| P00973 | OAS1 | 2 | 7.34 |
| O43524 | FOXO3 | 2 | 5.24 |
| P41182 | BCL6 | 2 | 4.93 |
| P17540 | CKMT2 | 2 | 3.38 |
| O43278 | SPINT1 | 2 | 0 |
| Q9BUP0 | EFHD1 | 1 | 0 |
| Q86WT6 | TRIM69 | 1 | 0 |
| Q13253 | NOG | 1 | 0 |
| P04798 | CYP1A1 | 1 | 0 |
| Q9NZQ7 | CD274 | 1 | 0 |
| O43490 | PROM1 | 1 | 0 |
| Q99705 | MCHR1 | 1 | 0 |
| Q9C091 | GREB1L | 1 | 0 |
| Q9ULB5 | CDH7 | 1 | 0 |
| O75360 | PROP1 | 1 | 0 |
| Q6NXR0 | IRGC | 1 | 0 |
| Q96GA7 | SDSL | 1 | 0 |
| P49908 | SEPP1 | 1 | 0 |
| P02100 | HBE1 | 1 | 0 |
| Q14693 | LPIN1 | 1 | 0 |
| P10966 | CD8B | 1 | 0 |
| P55008 | AIF1 | 1 | 0 |
| P59923 | ZNF445 | 1 | 0 |
| Q8TD94 | KLF14 | 1 | 0 |
| Q8IXN7 | RIMKLA | 1 | 0 |
