# Supplementary material for: Immunobiotic Lactobacillus jensenii TL2937 Alleviates Dextran Sodium Sulfate-Induced Colitis by Differentially Modulating the Transcriptomic Response of Intestinal Epithelial Cells
Source: Front Immunol. 2020 Sep 17;11:2174. doi: 10.3389/fimmu.2020.02174 (PMC7527445; doi:10.3389/fimmu.2020.02174)
Supplement: Supplementary Table 4 — List of differentially expressed genes induced by immunobiotic Lactobacillus jensenii TL2937 in porcine intestinal epithelial (PIE) stimulated with dextran sodium sulfate (DSS). [file Table_4.docx]

**Supplementary Table 4.**

| **Probe Name** | **Gene Symbol** | **DSS** | ***Lactobacillus jensenii* TL2937** | **Gene Name** |
| --- | --- | --- | --- | --- |
| A_72_P142476 | AMBP | NA | 4.325 | alpha-1-microglobulin/bikunin precursor |
| A_72_P035338 | AMCF-II | 1.604 | 1.260 | alveolar macrophage-derived chemotactic factor-II |
| A_72_P449564 | BD129 | -1.036 | NA | beta-defensin 129 |
| A_72_P474564 | CADM3 | NA | -1.349 | cell adhesion molecule 3 |
| A_72_P213482 | CCL11 | 1.058 | NA | chemokine (C-C motif) ligand 11 |
| A_72_P223332 | CCL4 | 1.790 | NA | chemokine (C-C motif) ligand 4 |
| A_72_P372628 | CCL5 | -1.511 | -1.480 | chemokine (C-C motif) ligand 5 |
| A_72_P223792 | CCR7 | -2.235 | -2.444 | chemokine (C-C motif) receptor 7 |
| A_72_P180896 | CD163L1 | 1.270 | NA | CD163 molecule-like 1 |
| A_72_P232472 | CD180 | 1.978 | NA | CD180 molecule |
| A_72_P559369 | CD209 | NA | -2.198 | CD209 molecule |
| A_72_P232617 | CD274 | NA | 1.078 | CD274 molecule |
| A_72_P443358 | CD28 | -1.917 | -1.956 | CD28 molecule |
| A_72_P185936 | CD3D | -1.104 | NA | CD3d molecule, delta (CD3-TCR complex) |
| A_72_P088461 | CD3G | 5.179 | NA | CD3g molecule, gamma (CD3-TCR complex) |
| A_72_P077421 | CD4 | NA | 1.082 | CD4 molecule |
| A_72_P544217 | CD40 | NA | 1.079 | CD40 molecule, TNF receptor superfamily member 5 |
| A_72_P303229 | CD40LG | NA | -1.031 | CD40 ligand |
| A_72_P474664 | CD5L | NA | 1.011 | CD5 molecule-like |
| A_72_P088196 | CD80 | NA | 1.460 | CD80 molecule |
| A_72_P474454 | CD84 | 1.219 | NA | CD84 molecule |
| A_72_P088456 | CD8B | NA | 1.900 | CD8b molecule |
| A_72_P001301 | CD96 | -1.508 | NA | CD96 molecule |
| A_72_P302604 | CFB | 2.313 | NA | complement factor B |
| A_72_P441514 | CSF3 | -2.079 | -1.696 | colony stimulating factor 3 (granulocyte) |
| A_72_P165266 | CXCL10 | 1.947 | 2.740 | chemokine (C-X-C motif) ligand 10 |
| A_72_P372543 | CXCL13 | -3.618 | -4.425 | chemokine (C-X-C motif) ligand 13 |
| A_72_P442032 | CXCL9 | 2.180 | NA | chemokine (C-X-C motif) ligand 9 |
| A_72_P088501 | CXCR4 | NA | -1.093 | chemokine (C-X-C motif) receptor 4 |
| A_72_P661857 | CXCR4 | NA | -1.053 | chemokine (C-X-C motif) receptor 4 |
| A_72_P232817 | CXCR6 | 1.670 | NA | chemokine (C-X-C motif) receptor 6 |
| A_72_P223517 | EGFR | -1.930 | NA | epidermal growth factor receptor |
| A_72_P739864 | EPCAM | NA | -1.287 | epithelial cell adhesion molecule |
| A_72_P444051 | F2R | NA | 1.112 | coagulation factor II (thrombin) receptor |
| A_72_P177461 | F3 | 1.068 | 1.271 | coagulation factor III (thromboplastin, tissue factor) |
| A_72_P467035 | F9 | -1.472 | NA | coagulation factor IX |
| A_72_P455318 | FGF23 | 1.084 | NA | fibroblast growth factor 23 |
| A_72_P706634 | FGFR2 | NA | 2.085 | fibroblast growth factor receptor 2 |
| A_72_P621557 | FGG | -1.464 | -1.536 | fibrinogen gamma chain |
| A_72_P598473 | FGL2 | NA | 1.222 | fibrinogen-like 2 |
| A_72_P409208 | GDF15 | NA | -1.298 | growth differentiation factor 15 |
| A_72_P232892 | IFN-DELTA-1 | -1.164 | NA | interferon-delta-1 |
| A_72_P494189 | IFN-DELTA-4 | 2.157 | 5.942 | interferon-delta-4 |
| A_72_P577723 | IGSF1 | -2.061 | -2.153 | immunoglobulin superfamily, member 1 |
| A_72_P440881 | IL12RB1 | -1.054 | -1.137 | interleukin 12 receptor, beta 1 |
| A_72_P004386 | IL15 | 1.297 | NA | interleukin 15 |
| A_72_P302974 | IL16 | NA | -1.129 | interleukin 16 |
| A_72_P444799 | IL1A | 1.308 | 1.317 | interleukin 1, alpha |
| A_72_P165276 | IL1A | 1.343 | 1.276 | interleukin 1, alpha |
| A_72_P146391 | IL2 | NA | 1.221 | interleukin 2 |
| A_72_P303129 | IL2RG | NA | 2.743 | interleukin 2 receptor, gamma |
| A_72_P352408 | IRF5 | NA | 1.414 | interferon regulatory factor 5 |
| A_72_P142591 | MAP3K6 | -1.023 | NA | mitogen-activated protein kinase kinase kinase 6 |
| A_72_P201327 | MAPK13 | NA | 1.070 | mitogen-activated protein kinase 13 |
| A_72_P414078 | MPO | -1.250 | -1.328 | myeloperoxidase |
| A_72_P027231 | MX2 | -1.410 | NA | myxovirus (influenza virus) resistance 2 (mouse) |
| A_72_P146491 | NCF1 | -1.001 | NA | neutrophil cytosolic factor 1 |
| A_72_P636902 | NCF2 | -2.444 | -2.530 | neutrophil cytosolic factor 2 |
| A_72_P490153 | NFKB2 | 1.149 | NA | nuclear factor of kappa light polypeptide gene enhancer in B-cells 2 (p49/p100) |
| A_72_P441199 | NOS2 | NA | -1.022 | nitric oxide synthase 2, inducible |
| A_72_P223657 | OAS1 | NA | 1.233 | 2'-5'-oligoadenylate synthetase 1, 40/46kDa |
| A_72_P232632 | PROC | -1.877 | -1.954 | protein C (inactivator of coagulation factors Va and VIIIa) |
| A_72_P035596 | SELE | 1.286 | 1.949 | selectin E |
| A_72_P165741 | SELL | 1.184 | NA | selectin L |
| A_72_P260597 | SLA-6 | -1.622 | NA | MHC class I antigen 6 |
| A_72_P672583 | SLA-DQB1 | -1.281 | NA | SLA-DQ beta1 domain |
| A_72_P561324 | SLA-DRA | -1.799 | NA | MHC class II DR-alpha |
| A_72_P626735 | SLA-DRB1 | -1.318 | NA | MHC class II histocompatibility antigen SLA-DRB1 |
| A_72_P185956 | SOCS3 | -1.212 | NA | suppressor of cytokine signaling 3 |
| A_72_P176391 | TFF2 | 2.011 | NA | trefoil factor 2 |
| A_72_P377788 | TGFA | NA | -1.182 | transforming growth factor, alpha |
| A_72_P077506 | TGFBR3 | -2.867 | NA | transforming growth factor, beta receptor III |
| A_72_P538206 | TNF | 1.196 | NA | tumor necrosis factor |
| A_72_P443413 | TNFRSF11B | 1.116 | NA | tumor necrosis factor receptor superfamily, member 11b |
| A_72_P473423 | TNFRSF11B | 1.210 | NA | tumor necrosis factor receptor superfamily, member 11b |
| A_72_P077441 | TNFSF4 | -3.482 | -3.857 | tumor necrosis factor (ligand) superfamily, member 4 |
| A_72_P118246 | VWA3A | -2.026 | -1.839 | von Willebrand factor A domain containing 3A |
